# Root Exudation of Primary Metabolites: Mechanisms and Their Roles in Plant Responses to Environmental Stimuli

**DOI:** 10.3389/fpls.2019.00157

**Published:** 2019-02-21

**Authors:** Alberto Canarini, Christina Kaiser, Andrew Merchant, Andreas Richter, Wolfgang Wanek

**Affiliations:** ^1^ Terrestrial Ecosystem Research, Department of Microbiology and Ecosystem Science, Research Network ‘Chemistry Meets Microbiology’, University of Vienna, Vienna, Austria; ^2^ Faculty of Science, Sydney Institute of Agriculture, The University of Sydney, Sydney, NSW, Australia

**Keywords:** root exudates, soil micro-organisms, root architecture, nutrient sensing, priming effect, mycorrhiza

## Abstract

Root exudation is an important process determining plant interactions with the soil environment. Many studies have linked this process to soil nutrient mobilization. Yet, it remains unresolved how exudation is controlled and how exactly and under what circumstances plants benefit from exudation. The majority of root exudates including primary metabolites (sugars, amino acids, and organic acids) are believed to be passively lost from the root and used by rhizosphere-dwelling microbes. In this review, we synthetize recent advances in ecology and plant biology to explain and propose mechanisms by which root exudation of primary metabolites is controlled, and what role their exudation plays in plant nutrient acquisition strategies. Specifically, we propose a novel conceptual framework for root exudates. This framework is built upon two main concepts: (1) root exudation of primary metabolites is driven by diffusion, with plants and microbes both modulating concentration gradients and therefore diffusion rates to soil depending on their nutritional status; (2) exuded metabolite concentrations can be sensed at the root tip and signals are translated to modify root architecture. The flux of primary metabolites through root exudation is mostly located at the root tip, where the lack of cell differentiation favors diffusion of metabolites to the soil. We show examples of how the root tip senses concentration changes of exuded metabolites and translates that into signals to modify root growth. Plants can modify the concentration of metabolites either by controlling source/sink processes or by expressing and regulating efflux carriers, therefore challenging the idea of root exudation as a purely unregulated passive process. Through root exudate flux, plants can locally enhance concentrations of many common metabolites, which can serve as sensors and integrators of the plant nutritional status and of the nutrient availability in the surrounding environment. Plant-associated micro-organisms also constitute a strong sink for plant carbon, thereby increasing concentration gradients of metabolites and affecting root exudation. Understanding the mechanisms of and the effects that environmental stimuli have on the magnitude and type of root exudation will ultimately improve our knowledge of processes determining soil CO_2_ emissions, ecosystem functioning, and how to improve the sustainability of agricultural production.

## Introduction

The process of carbon (C) allocation and its adaptability is vitally important for plants to successfully respond to changing environmental conditions. Indeed, maximizing the trade-offs between investments and returns in terms of energy, water, C and nutrients will ultimately determine a plant’s growth, survival, and interaction with its microbiota. External stresses such as competition, nutrient, and/or water limitation cause a series of responses in plants that modify C allocation to maximize the gain of limiting resources. A plethora of research has shown that plant belowground C allocation is tightly connected to water and nutrient cycles (Cheng et al., 2010; McCormack et al., 2015; Gill and Finzi, 2016; Ledo et al., 2018). Yet, a major component of belowground C allocation, namely the process of root exudation, remains poorly understood. It remains unclear why and how plants invest up to 20–40% of their photosynthetically fixed C in root exudates (Badri and Vivanco, 2009). Current ecological theories link root exudation to a benefit for plants *via* stimulation of beneficial micro-organisms (e.g., symbionts), promoting nutrient acquisition and enabling recognition between self-roots and neighbor-roots (Ortíz-Castro et al., 2009; Dijkstra et al., 2013; Yin et al., 2013; Depuydt, 2014; Mommer et al., 2016; Meier et al., 2017). However, while some root exudates, such as bioactive secondary compounds, are actively exuded from roots through energy-consuming primary or secondary active transporters (Sasse et al., 2018), the majority of them are represented by primary metabolites (mainly sugars, amino acids, and organic acids) in which many studies suggest to be passively lost from the root at the meristematic root apex (McCully and Canny, 1985; Darwent et al., 2003; Jones et al., 2009). In this context, several fundamental questions emerge:

1. What is the mechanism driving root exudation of primary metabolites?
2. Do plants have control over this process through adjustments in plant source-sink dynamics and efflux carrier expression and what are the consequences for root growth?
3. Can plants sense the concentrations of exuded and/or soil-borne metabolites?
4. Are these metabolites somehow involved in nutrient foraging through root exudation?

In this manuscript, we propose a conceptual framework built upon recent advances in different disciplines of ecology and biology linking plants with the soil environment. Here, we focus on primary metabolites that are exuded to the soil (sugars, amino acids, and organic acids) and for which specific concentration gradients influence their root exudation. The transient concentrations of these metabolites in the root tip serve as a cue for environmental sensing by plants and signaling between roots and shoots to modify root growth and carbon allocation. Our framework suggests that root exudates are used by the plant to complement the function of nutrient transporters in sensing nutrient availability and in signaling nutrient supply relative to demand. This process therefore optimizes root growth to facilitate effective nutrient foraging and possibly to sense competing neighbors. Also, given that a vast proportion of root exudation is driven by diffusion, soil micro-organisms will play an important role in driving concentration gradients outside the root tip, thus affecting exudation rates. We will utilize examples from studies on root exudation and plant nutrient acquisition strategies to support our framework and then analyze ecosystem scale impacts to highlight the relevance of the proposed mechanism in contributing to soil organic matter decomposition and CO_2_ emissions, plant community assemblage processes, and plant productivity.

## Facilitated Diffusion-Driven Root Exudation and the Role of Micro-Organisms

The majority of root exudation is localized at the root tip (McCully and Canny, 1985; Jaeger et al., 1999; Doan et al., 2017; Sasse et al., 2018). The root tip is the first plant part to explore new soil environment and plays a crucial role in root responses to environmental stimuli. We will now illustrate how primary metabolites can be released from the root tip and how microbes interact with this process. While this manuscript will cover primary metabolites, it is important to highlight that roots also exude a wide range of secondary metabolites and further release high molecular weight compounds into the soil through rhizodeposition, most importantly root border cells and mucilage. These types of rhizodeposits can serve important functions in the soil. For example, they represent an important nutrient source for rhizosphere microbes and influence root-microbe and root-symbiont relations (for example, Hawes et al., 2002, 2016; el Zahar Haichar et al., 2014; Ahmed et al., 2018).

### Release of Primary Metabolites at the Root Tip

**Figure 1:**
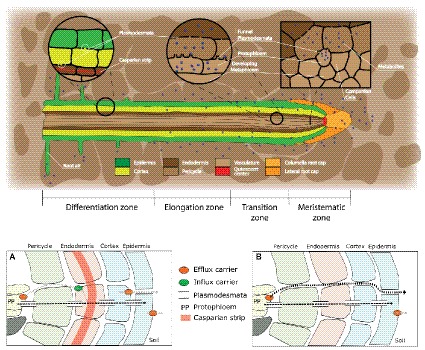
Root structure and areas of root exudation. The upper figure represents the longitudinal section of a root. Tissues are indicated in different colors for the different zones of the root (listed at the bottom). The two circles focus on two distinct zones, a differentiated vs. an undifferentiated area, to show the presence of a Casparian strip and low abundance of plasmodesmata in the differentiated area (left circle), and the presence of funnel plasmodesmata in the undifferentiated area (right circle). The square represents a cross section close to the meristematic area where root exudation is the highest. The lower figures represent a schematic representation of solute movement sites from phloem unloading to the soil environment, either in the differentiation zone **(A)** or in the undifferentiated root tip **(B)**. **(A)** Solutes move both through the symplastic and apoplastic pathways, but then they are re-uptaken into the cytoplasm as the Casparian strip limits the apoplastic pathway. Only the cortex and epidermis are responsible for the flux of metabolites into the apoplast and consecutively into the soil (root exudation). Cortex and epidermis represents the major control point for root exudation. **(B)** At the phloem unloading site, both symplastic and apoplastic pathways are used. Because of the lack of a Casparian strip solutes can move out of the root (root exudation) through both the apoplastic and the symplastic pathway.

Root exudation is a complex phenomenon encompassing processes that drive C transport to roots and exudation from roots to soil. The long distance transport of C produced in source organs takes place in the phloem, through the widely accepted Munch’s pressure-driven mechanism of phloem flow (Münch, 1930). According to this mechanism, phloem metabolites are transported by a difference in turgor between sink and source organs generated by concentration gradients, which are determined by source-sink activities (De Schepper et al., 2013). Recent experiments support this hypothesis (Knoblauch et al., 2016) and have shed light on how metabolites are unloaded from the phloem to the actively growing root tip (Ross-Elliott et al., 2017), knowledge which is essential to understand root exudation. Specifically, Ross-Elliott et al. (2017) showed that in *Arabidopsis,* phloem unloading occurs through plasmodesmata in a convective way (combination of mass flow and diffusion). During unloading, low-molecular-weight solutes and proteins are diverted into the phloem-pole pericycle, a tissue connected to the protophloem by a unique class of “funnel plasmodesmata” (Figure 1). While proteins are released in discrete pulses (referred to as “batch unloading”) and remain restricted to the phloem-pole pericycle, low-molecular-weight organic solutes are unloaded without restrictions and move out of the phloem-pole pericycle. The discovery made by Ross-Elliott et al. (2017) is very important in connection to root exudation at the root tip. Indeed, they demonstrate that this area is the principal route for all solutes to be unloaded and that they will move toward the surrounding cells through diffusion (Ross-Elliott et al., 2017) because of the high degree of plasmodesmatal connections in this area (Figure 1; Rutschow et al., 2011).

### Movement of Primary Metabolites Outside the Root Tip

While metabolites can move quite freely through the symplastic pathway, in order to be excreted to the soil environment, they need to pass through at least one plasma membrane to reach the apoplast. The plasma membrane is permeable to gas and to small molecules (such as urea or glycerol), while it is virtually impermeable against larger, uncharged polar molecules (e.g., glucose) and against all charged molecules including ions (Yang and Hinner, 2015). Therefore, these molecules only transit the plasma membranes through specific transmembrane proteins, which form small pores through the lipid bilayer, allowing polar or charged molecules to cross the membrane without interacting with the hydrophobic fatty acid chains of the membrane phospholipids (Sasse et al., 2018).

**Figure 2:**
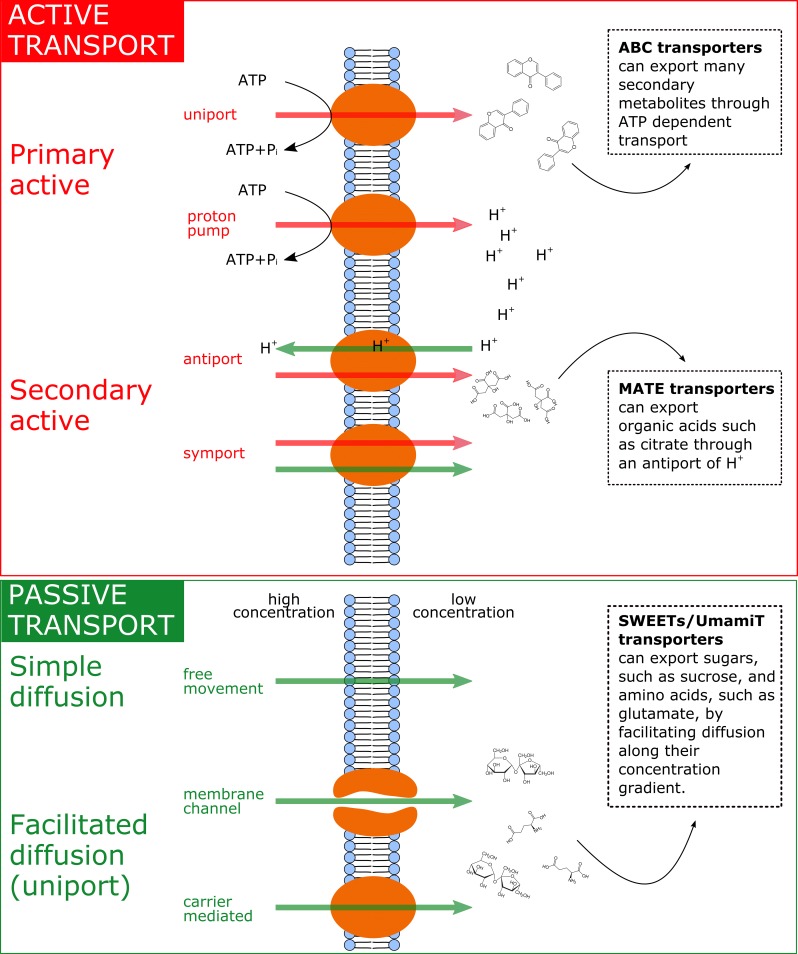
Summary of the main exudation mechanisms through the plasmamembrane at the root tip. The top panel represents active transport mechanisms, either primary active (direct consumption of ATP) or secondary active (e.g. coupled to H^+^ pumps that actively consume ATP). The bottom panel represents passive transport mechanisms, which allow diffusion following electrochemical gradients. Red arrows represent movement of solutes against their electrochemical gradient, while green arrows represent movement following their electrochemical gradient. On the right side of the figure, examples of membrane transporters allowing movement of primary metabolites are provided.

It was recently discovered that the efflux of sugars, organic acids, and amino acids is mediated through specific efflux carriers and channels that may allow a fine tuning of the exudation flux through up/downregulation of their gene expression or at the level of post-translational modification (Badri et al., 2008). Some of these efflux transporters of primary metabolites have been characterized, e.g., for amino acids [UMAMIT transporters (Okumoto and Pilot, 2011; Moe, 2013; Besnard et al., 2016; Dinkeloo et al., 2017), CAT transporters (Yang et al., 2010), GDU transporters (Pratelli et al., 2010)], sugars [probably belonging to the SWEET transporter family (Williams et al., 2000; Chen et al., 2015; Manck-Götzenberger and Requena, 2016)], and organic acids [ALMT/malate and MATE/citrate transporters (Meyer et al., 2010; Mora-Macías et al., 2017)]. Most of these transporters are not primary or secondary active and therefore are not directly coupled to ATP hydrolysis or to ATP-dependent H^+^ pumps and H^+^ antiports (see Figure 2). The exception to this is the ATP-dependent ABC transporters for secondary compounds (Badri et al., 2009) and MATE/citrate transporters that possess H^+^-coupled antiport activity (Meyer et al., 2010). All other transporters were shown not to be coupled to ATP hydrolysis or to H^+^ cotransport. The common denominator of most of the primary metabolite transporters is that they are substrate-specific facilitators, facilitating the diffusion of primary metabolites across the plasma membrane *via* transmembrane carriers along the concentration gradient, i.e., from high intracellular to low extracellular concentrations. The exact genes governing the synthesis and abundance of the UMAMIT and SWEET transporters involved in root amino acid and sugar exudation have not yet been identified, and relatively little is currently known regarding the plant demand for nutrients and environmental and edaphic factors that regulate their gene transcription. Only recently, pathogen-driven activation of SWEET expression was shown to increase glucose efflux into the root apoplast (Chen et al., 2015), and Al^3+^ toxicity or P deficiency triggers exudation of malate by ALMT upregulation in roots (Ma et al., 2001; Kochian et al., 2005; Mora-Macias et al., 2017). Identifying the responsible efflux carriers for the most abundant primary metabolite components of the exudates at the root-soil interface will revolutionize our understanding of the regulation of root exudation (Sasse et al., 2018).

Once metabolites have moved out of the phloem cell plasma membrane, and they may follow the apoplastic transport pathway. Indeed, a recent study suggests that in immature wheat roots, carbon containing compounds would circulate from the stele to the cortex through an apoplastic pathway and then be used by microbes in the soil environment (Vidal et al., 2018). In the apoplast, the diffusion process would regulate the flux from roots to the soil environment, without any obstacle represented by plasma membranes. This could be very important at the root tip. Indeed, while at distance from the meristem, in the differentiation zone, endodermis cells develop a barrier against apoplastic diffusion (Naseer et al., 2012; Somssich et al., 2016), the meristem lacks an apoplastic barrier (“Casparian strip”; Figure 1). Also, epidermis cell walls in the mature area of root tip of Arabidopsis showed more than 10 times slower apoplastic diffusion rates as compared to the elongation zone (Kramer et al., 2007). It would therefore be interesting to investigate the presence of a root hot spot, where carbon containing compounds are released to the apoplast (Figure 1). If indeed a hot spot could be identified close to the area in which phloem unloading occurs, this would indicate that the apoplastic pathway is a major contributor to root exudation and establishes a close link between the phloem and the soil environment. Indications of an apoplastic pathway are provided by studies showing that bacteria mostly distribute along the axial grooves in between neighboring root epidermal cells (Watt et al., 2006; Schmidt et al., 2018). However, these results could not exclude a direct loss from epidermal cells (Jones et al., 2009) or could indicate that grooves simply represent areas of physical protection for micro-organisms (Schmidt et al., 2018). Indeed, the specific pathway by which solutes move from the unloading area to the external medium is still matter of debate, and a switch from symplastic to apoplastic pathways with root development was also shown (Godt and Roitsch, 2006). More studies on the spatial and temporal dynamics of solute fluxes at the root tip are therefore necessary to improve our understanding of the plant control over the root exudation process.

From reasons outlined above, we therefore postulate that the anatomy of the root tip promotes the diffusion of metabolites (or offer less resistance) from the root apex to the external soil environment and thereby ultimately determines the concentration gradient-dependent outward flux of solutes, i.e., the rate of root exudation. Root tip exudation will be highly dependent on diffusion rates and therefore on concentration gradients between rhizodermal cells and the soil environment. This process will ultimately draw metabolites from the phloem and indicate a close coupling between photosynthetic activity and root exudation, which is strongly influenced by the consumption and transformation of metabolites by microbes in the rhizosphere. This hypothesis is supported by empirical studies showing: (1) localized exudation at the root tip level (McCully and Canny, 1985; Jaeger et al., 1999; Doan et al., 2017); (2) correlation between exudates and root growth (Lucas García et al., 2001); (3) coupling of exudation and photosynthesis (Kuzyakov et al., 2003; Mencuccini and Hölttä, 2010); and (4) reduction of root exudation when plants are allocating resources to reproductive organs instead of root growth (Prikryl and Vancura, 1980; Badri and Vivanco, 2009).

However, it is important to acknowledge two facts. First, many compounds are also exuded by active transport against the concentration gradient (citrate and secondary compounds). Second, while facilitated diffusion-driven root exudation is “passive” in its nature following the concentration gradient from high cytoplasmic to low external concentrations in the soil solution (because diffusion does not require plants to spend energy), plants can still control this process in different ways:

1. Induction/repression of gene expression and post-translational modification of efflux carriers for sugars, amino acids, and organic acids;
2. Possible re-uptake of exuded organic solutes and inorganic nutrients through uptake transporters and their regulation as in (1);
3. Changes in source/sink dynamics including processes at phloem loading sites, import of solutes into root cells and compartmentalization within root epidermal cells (vacuolar loading/sequestration of solutes), and changes in meristematic activity (e.g., root meristem exhaustion).

The above-mentioned mechanisms come at an energy cost for the plant, through secondary active re-uptake of metabolites and by energy-intensive biosynthesis of the efflux and influx carrier proteins. Therefore, we suggest to avoid the use of “passive exudation” to define the root exudation process.

### The Role of Micro-organisms in Facilitated-Diffusion Driven Root Exudation

Based on the diffusive nature of the exudation process as described above, we also propose that microbial utilization and metabolism play a pivotal role in modulating concentration gradients right outside root tips, thereby constituting a soil sink and promoting root exudation. Indeed, it was found that when micro-organisms were present in plant growth solution, exudation was enhanced as compared to axenic solutions (Biondini et al., 1988; Jones and Darrah, 1993; Meharg and Killham, 1995; Groleau-Renaud et al., 2000; Phillips et al., 2004; Vranova et al., 2013; Pii et al., 2015b). Some studies concluded that concentration gradients drew solutes out of the roots, although re-absorption of metabolites has also been indicated (Jones and Darrah, 1993; Warren, 2015, 2016). Enhancement of exudation was also observed in the absence of micro-organisms but through reduction of the external concentration in axenic solution by using pure water to collect exudates (Groleau-Renaud et al., 2000; Valentinuzzi et al., 2015) or by simulating the effect of removal of metabolites by regularly changing the growth solution. Additionally, the presence of micro-organisms in the growth solution altered plant C allocation to roots and root/shoot ratios (Jones and Darrah, 1993; Groleau-Renaud et al., 2000). In a more recent experiment, activated charcoal was used to evaluate the removal of exudates from the growth medium, showing that the absence of root exudates from the root surroundings caused a re-programming of the whole root system architecture in *Arabidopsis* (Caffaro et al., 2011). It is known that there is a tight relationship between root exudates and microbial diversity (Eisenhauer et al., 2017), but it is still unknown at what level they influence each other. Microbial diversity is affected by exuded primary metabolites (Shi et al., 2011; Steinauer et al., 2016), and at the same time, it affects specific concentration gradients outside the root through preferential microbial utilization of plant exudates. Also, soil microbes can release compounds such as 2,4-diacetylphloroglucinol and zearalenone that stimulate root exudation of primary metabolites, such as amino acids (Phillips et al., 2004). It was suggested that current models depicting C pools/fluxes and vegetation dynamics are mainly structured as “source driven” models. It was argued that the control of environmental cues (e.g., temperature, water, and nutrient availability) on meristematic activity (sink capacity) are pivotal because they mostly become limiting to plant growth before the source capacity represented by photosynthesis responds (Fatichi et al., 2014; Körner, 2015). While source-sink dynamics are increasingly recognized as fundamental in understanding plant C allocation, micro-organisms should also be considered as a strong sink of plant photosynthates, thereby promoting root exudation (Farrar and Jones, 2000; Savage et al., 2016).

### Interactions Between Mycorrhizal Associations and Root Exudates

While our knowledge on both the function and magnitude of root system architecture, nutrient sensing, and root exudation has significantly increased, it remains unclear how mycorrhizae are involved in these processes. Indeed most laboratory studies on root exudation did not include mycorrhizal associations, while mycorrhizal fungi colonize the roots of about 90% of all land plant species. The establishment of mycorrhizal association is mediated by controlled exudation and sensing of specific secondary metabolites by the roots (Parniske, 2008; Bonfante and Requena, 2011; Martin et al., 2017). However, once the association is established, the major flow of C from plants to the fungus consists of recently photoassimilated sugars and fatty acids transferred across the symbiotic interface. Whether or not this major diversion of belowground C flow alters root exudation rates of plants qualitatively or quantitatively is not known.

In the colonization phase, plant roots communicate in a complex way with their potential mycorrhizal partners *via* exchange of chemical signals. Different mechanisms exist for arbuscular mycorrhizae (AM), which are the most widespread form of mycorrhizal association (formed by 74% of all vascular plant species), and ectomycorrhizal (EM) symbiosis, which is dominant in temperate and boreal forest ecosystems (for more information, see, for example, Parniske, 2008; Smith and Read, 2008; Brundrett, 2009; Smith and Smith, 2011; Gutjahr and Parniske, 2013). In either case, once the mycorrhizal association has been formed, mycorrhizal fungi receive a substantial fraction of the plant belowground C flux as their sole energy and C source while delivering nutrients to the plant in return (Van Der Heijden et al., 2015). How and where this C is transferred from plant roots to their mycorrhizal partners differs strongly between different types of mycorrhizal association. In the AM symbiosis, fungal hyphae grow and form arbuscules inside cells of the root cortex along the entire length of plant fine roots. A substantial amount of recently assimilated C is diverted from the phloem and allocated into intraradical hyphae and arbuscules and further into the fungal extraradical hyphal network (Drigo et al., 2010; Kaiser et al., 2015; Paterson et al., 2016). Whether this pathway, which represents a substantial drawing of C “upstream” of the root tip, consequently reduces root exudations at the tip is not known. It has been shown, however, that AM colonization increases plant photosynthetic rates (Grimoldi et al., 2006; Johnson et al., 2015), which may reflect increased demand from increased belowground C transfer to the fungi. Also AM colonized plants show increased sugar transport to roots. It has therefore been suggested that this increased sugar flux may cause lateral root formation (as discussed in “The role of organic acids and sugars in P acquisition strategies” section for P sensing), which is typical of AM colonized plants (Fusconi, 2014). Ectomycorrhizal fungal hyphae, in contrast, do not grow inside root cells but form a tight mantle completely covering the root tips, with fungal hyphae growing in between the outer layers of root epidermal cells, forming the Hartig Net (Smith and Read, 2008). Within the Hartig Net, plant and fungal cells are separated by an apoplastic space that serves as an exchange zone for C released by plant cells and nutrients released by the fungus (Nehls, 2008). This highlights the potential of plant cells to use the apoplastic space in the root cortex as exudation zone, as opposed to direct exudation from epidermal cells (Jones et al., 2009). In contrast to AM and other endomycorrhizal associations, EM may effectively reduce or even stop root exudates from being transferred into the soil due to the formation of the tight hyphal mantle “sealing” the root tip. A recent study has shown that exudation rates of AM vs. EM-colonized roots only changed when plants were drought stressed (Liese et al., 2018). To what extent EM may affect root exudations compared to AM or if part of the plant-derived C is exuded from the fungal cells at the outside of the hyphal mantle is still not known.

Plant-derived C transferred to the plant’s mycorrhizal partner is quickly transported into the hyphal network extending into the soil. *In vitro* studies have shown hyphal exudation of carbohydrates and amino acids (Bharadwaj et al., 2012; Zhang et al., 2018). Therefore, it has been hypothesized that fungal hyphae exude this labile C at root-distant places into the soil, where it may stimulate decomposition of organic matter by free-living soil saprotrophs and consequently increases nutrient availability (Jansa et al., 2013; Kaiser et al., 2015; Meier et al., 2015; Paterson et al., 2016). In other words, hyphal exudation could trigger (analogous to the rhizosphere priming effect) a hyphosphere priming effect. Indications for hyphosphere priming and actual transfer of recently photoassimilated C *via* mycorrhizal hyphae to soil bacteria has been shown for AM associations (Toljander et al., 2007; Drigo et al., 2010; Cheng et al., 2012; Kaiser et al., 2015; Paterson et al., 2016) and hyphal-bacterial transfer of plant-derived C recently also for EM associations (Gorka et al., 2019).

Despite the overwhelming presence of mycorrhiza in terrestrial ecosystems, they are neglected in most root exudate research. One reason for them being overlooked may be attributed to common model plants, like *Arabidopsis*, belonging to the 5–10% of vascular plant species that do not form mycorrhizal associations. Studies that integrate mycorrhizae into “traditional” rhizosphere research are urgently needed to close this research gap and improve our understanding of the interactions between mycorrhiza, root exudation, and rhizospheric processes.

## Sensing and Signaling of Nutrients by Concentration Gradients

It is not a new concept that root exudates are used to sense and interact with the abiotic and biotic components of the soil environment (Mommer et al., 2016; Martin et al., 2017) and to aid in nutrient acquisition (Dakora and Phillips, 2002; Ström et al., 2002). However, studies of such interactions often addressed only specific compounds (e.g., secondary metabolites) and therefore leave unexplained possible functions of the largest proportion of root exudates, i.e., primary metabolites. We propose that this efflux from the actively growing root cells regulates the temporal concentrations of primary metabolites inside the root tip, many of which have the double function of metabolite and signaling molecule (Häusler et al., 2014). Any changes that modify the influx and/or the efflux of metabolites at the root tip can have cascading effects on the root system architecture, with the latter being defined as the spatial configuration of the root system. Root system architecture is a key factor in determining the ability of a plant to respond to nutrient hotspots, thereby maximizing the acquisition of soil resources (Khan et al., 2016). It is well known that root architecture is genetically determined, but that the chemical and physical characteristics of the soil environment ultimately shape root growth, especially in response to soil nutrients (Lynch, 1995; López-Bucio et al., 2003). Recent advancements in studies of nutrient uptake and root system architecture responses to nutrient-rich patches have strongly advanced our understanding of the fine tuning between roots and shoots to control root growth. Here, we highlight examples of how common metabolites present in root exudates can be sensed by the root to modify root system architecture responses to environmental cues. We place particular focus on the two main nutrients limiting plant growth: nitrogen (N) and phosphorus (P).

### The Role of Amino Acids in N Acquisition Strategies

Plants are highly flexible in adapting to changing conditions of soil N availability (Giehl and von Wirén, 2014), which implies that they can sense N in the soil. The main examples of this flexibility come from studies on nitrate (NO_3_^−^) uptake and sensing. Nitrate possesses the strongest signaling effects on root system architecture, which seems to be even more important than its nutritional function (Sun et al., 2017). For example, under low NO_3_^−^ supply, plants tend to adopt a “foraging strategy” with lateral root proliferation to enhance nutrient access. Root responses to NO_3_^−^ are mediated by a family of transporters (mainly NRT2 and NRT1), which can modulate the transport of auxin (and other plant hormones and signal molecules) to regulate the development of root system architecture. The regulatory network connecting nitrate transporters to changes in root system architecture is complex in nature due to the multi-stage root branching process, the dependence on nitrate levels in soil (high or low) and sometimes demonstrating antagonism between signaling pathways. We refer the reader to more in-depth reviews on this topic (see, Sun et al., 2017; Forde, 2014). While in high fertility soil microsites, NO_3_^−^ tends to dominate the available soil N pool, and in low fertility soil microsites, amino acids become dominant (Schimel and Bennett, 2004; Rothstein, 2009).

Amino acids are one of the main components of root exudates, are omnipresent in the soil environment at low concentrations, and might therefore represent important triggers of plant responses to changing N availability in soil. Indeed, it was demonstrated that amino acids are the main indicator of the N status of plants, which is important for the regulation of plant N uptake (Pal’ove-Balang and Mistrík, 2002; Nacry et al., 2013; Forde, 2014; Kellermeier et al., 2014; Gent and Forde, 2017). For example, it was found that amino acid export from the leaf to the phloem depends on the N level belowground (Caputo and Barneix, 1997). Also, in a split-plot experiment, N-deprived roots received significantly lower amino acid concentrations with altered composition from the phloem compared to N-supplied roots, which had similar amino acid composition in the phloem flow compared to the control (Tillard et al., 1998). Furthermore, amino acids represent a potential source of N for plants (Forsum et al., 2008; Jämtgård et al., 2008; Näsholm et al., 2009; Inselsbacher and Näsholm, 2012), and recent studies have demonstrated that amino acids play an important role in shaping root system architecture and nutrient foraging. For example, plants shut down nitrate uptake when amino acids are present in the growth medium at relatively high concentrations (Padgett and Leonard, 1996; Aslam et al., 2001; Henry and Jefferies, 2003; Dluzniewska et al., 2006). More specifically, many amino acids can induce repression of NRT2.1 gene expression, which is an important transporter and sensor of nitrate (Nazoa et al., 2003). On the other hand, NO_3_^−^ was shown to have no effect on the root uptake of the amino acid glycine (Gioseffi et al., 2012).

**Figure 3:**
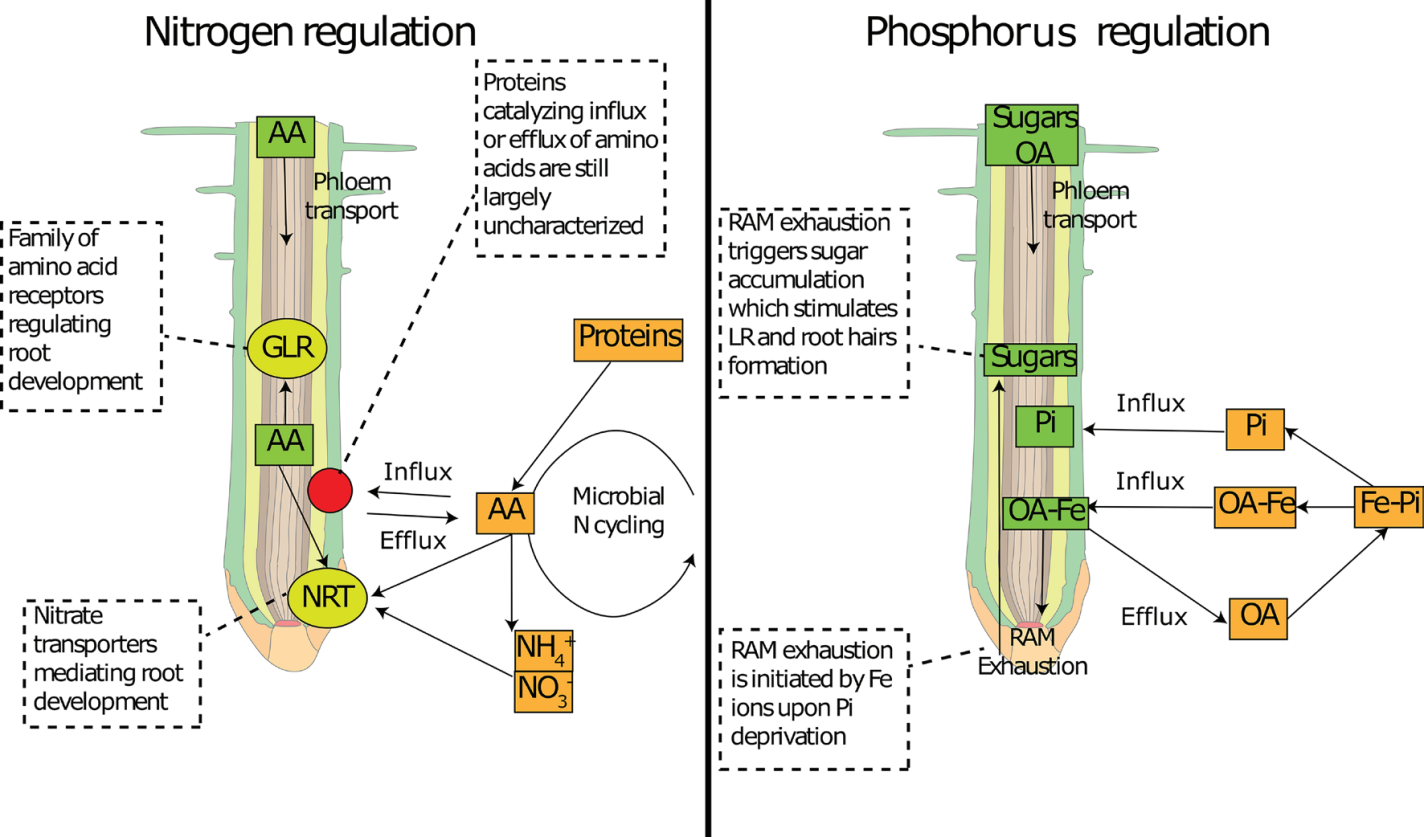
Involvement of common metabolites in nutrient sensing at the root tip. Schematic representation of how common primary metabolites allows nutrient sensing at the root tip for nitrogen (left panel) and phosphorus (right panel). Amino acid concentration can be sensed by specific receptors (GLR) and can interfere with N transporter expression (NRT). Amino acid concentration inside the root tip depends on the delivery from the phloem and on root exudation, the latter of which depends on concentration gradients mediated by soil micro-organisms. Organic acids can deliver Fe to the root tip, which activates the RAM exhaustion and temporarily increases sugar concentrations. Sugars can be sensed at the root tip and activate changes in root architecture. AA: amino acids; OA: organic acids; Pi: inorganic phosphorus; RAM: root apical meristem; NRT: nitrate transporters; GLR: glutamate-like receptors.

Among the 20 proteinogenic amino acids, the amino acid L-glutamate has been proven to cause the most intense responses in root system architecture in *Arabidopsis thaliana* (Forde, 2014). However, many other amino acids can cause similar responses in root system architecture. It has been suggested that it is not a single amino acid, but their overall concentration regulates these cell responses (Padgett and Leonard, 1996; Aslam et al., 2001). Glutamate acts in a way similar to auxin (indole-3-acetic acid, which is known to influence root system architecture and many other aspects of plant biology). These compounds, added exogenously to plant cells, elicit rapid membrane depolarization and a transient increase in [Ca^2+^]_cytosol_ (Zimmermann et al., 1999; Dennison and Spalding, 2000; Ni et al., 2016). Therefore, glutamate could create an electrical signal similar to what is observed in synaptic transmission processes in mammals (Baluška and Mancuso, 2013). While auxin can cause responses in roots at very low concentrations, the glutamate response requires higher concentrations of about 10^−5^ M compared to 10^−11^ M of auxin (Evans et al., 1994). The difference between the two molecules may lay in the fact that glutamate fulfills the dual role of primary metabolite and signal molecule, and physiological concentrations have to be maintained for metabolic activity in cells (Forde and Lea, 2007). Glutamate is also one of the most abundant amino acids in soils in the pool of dissolved organic N (Paynel et al., 2001). While in soil solution, glutamate concentrations are lower than 10 μM (Friedel and Scheller, 2002; Jones et al., 2005), around the root tip and in areas close to decomposing organic residues they are expected to be several-fold higher (Walch-Liu et al., 2006b). In agar plate experiments, locally high glutamate concentrations slowed primary root growth and encouraged root branching behind the primary root tip (Walch-Liu et al., 2006a). It has been hypothesized that this localized increase in root branching at the root tip would lead to increased precision of root placement within an organic N-rich patch (Walch-Liu et al., 2006a; Forde and Lea, 2007), which would represent a foraging response particularly important in low-fertility soils of temperate regions where rates of N mineralization may be low (Schimel and Bennett, 2004; Rothstein, 2009; Forde, 2014). Indeed, glutamate possesses all the characteristics to make it an important signaling molecule. First, plants possess effective mechanisms for glutamate re-absorption. Six members of the Arabidopsis AAP family (AAP1–AAP6) have been shown to catalyze the low affinity root uptake of a broad range of amino acids, including glutamate (Fischer et al., 2002), and the LHT1 gene appears to encode a high-affinity glutamate influx system (Hirner et al., 2006). Also, a ubiquitous family of receptors, termed glutamate receptor-like genes (GLR) (Teardo et al., 2011; Tapken et al., 2013; Vincill et al., 2013), are involved in amino acid sensing throughout the whole plant (Forde, 2014). The 20 GLR genes in *Arabidopsis* are differentially expressed in the plant at least at the mRNA level, and in roots, all 20 have been found to be expressed (Chiu et al., 2002; Roy et al., 2008). Studies on GLR knockout mutants have shown that in rice, the OsGLR3.1 gene positively regulates cell proliferation in the root apical meristem (Li et al., 2006). A knockout mutation of AtGLR3.2 and AtGLR3.4 genes affected lateral root primordia, suggesting a possible negative regulation of lateral root initiation by GLR (Vincill et al., 2013). Interestingly, expression analysis of the AtGLR3.2 and AtGLR3.4 proteins revealed that in the root they are primarily targeted to the phloem, particularly to the distal parts of mature phloem cells, in the vicinity of the sieve plates (Vincill et al., 2013). At least some members of the GLR receptor family that are expressed in roots are now known to act as amino-acid-gated Ca^2+^ channels with broad ligand specificity (Vincill et al., 2012). Moreover, we mentioned that amino acid loading into the phloem depends on the plant N level (Caputo and Barneix, 1997), and glutamate is commonly found in phloem sap. Thus, it was hypothesized that glutamate sent from shoots could be the signal to communicate plant N status to the roots (Forde and Lea, 2007). Therefore, given that glutamate can be sensed in the root tip phloem (Vincill et al., 2013) and translated into a signal affecting the root system architecture (Walch-Liu et al., 2006a; Forde and Lea, 2007), any change in influx or efflux of glutamate at the root tip will likely affect root development (Figure 3). While this highlights the specificity in the glutamate response, other amino acids have been shown to trigger changes in root system architecture, such as D-serine (Forsum, 2016). Also, most of the studies were carried out on *Arabidopsis*, while other plant species have shown different responses to glutamate (Domínguez-May et al., 2013; Kan et al., 2017). Our understanding of the effects of different amino acids on root system architecture and the mechanisms underlying different plant responses to glutamate therefore remain incomplete.

In low fertility soils where amino acids are the dominant form of N (Rothstein, 2009), they may represent a major driver of root nutrient foraging strategies. However, in high fertility soils, inorganic forms of N dominate and it is unclear what role amino acids may play in nutrient foraging under such conditions. It was shown that exudation rates of sugars, amino acids (glycine), organic acids, and phenolics increased with inorganic N addition rates increasing from 0 to 160 mg N L^−1^ in hydroponics (Zhu et al., 2016). Uptake of different N forms by plant roots can also affect root exudation patterns as, for example, plant ammonium nutrition strongly promotes sugar exudation compared to nitrate-grown plants and switching plants from ammonium to nitrate nutrition in hydroponics rapidly downregulated sugar efflux by 30-fold (Mahmood et al., 2002). Therefore, plant N nutrition, either in organic or in inorganic form, by having a strong effect on root exudation patterns might interact with expression of N uptake carriers and root system architecture in a way that is currently difficult to predict.

### The Role of Organic Acids and Sugars in P Acquisition Strategies

Root responses to P appear to play a major role in root system architecture compared to other nutrients (Kellermeier et al., 2014). Phosphorus deficiency causes drastic changes in root system architecture, generally by increasing the number of root hairs and lateral roots to increase the root surface area and thereby acquisition of P, which has very limited mobility in soil (Nadira et al., 2016). For example, many plants growing in extreme P limited environments have developed specialized root structures to release higher amounts of organic acids: the cluster roots or proteoid roots (Shane and Lambers, 2005; Lambers et al., 2006). Interestingly, the release of organic acids from cluster roots of Proteaceae and Fabaceae and also from roots of other plants is associated with young parts of the root system, such as the root apex (Shane and Lambers, 2005; Ryan et al., 2014). Rates of citrate efflux are the highest during the photoperiod, and no correlation with the activity of enzymes involved in citrate biosynthesis in roots was found (Watt and Evans, 1999). A primary requirement to initiate the root response is to establish root contact with areas of very low P. Contact of the root tip with a low-P_i_ medium results in the arrest of primary root growth, including reduced cell elongation, cell division, and meristematic activity, regardless of shoot P_i_ status (Chevalier et al., 2003; Ticconi and Abel, 2004; Svistoonoff et al., 2007). A recent study using mutants lacking a root cap revealed that while the root cap is responsible for about 20% of total P absorption in *Arabidopsis*, it is not involved in adaptations of root system architecture (Kanno et al., 2016). An important mechanism necessary for the root architecture response to P deficiency was found to be root exudation of malate (Mora-Macías et al., 2017). Indeed organic acids such as malate and citrate are important compounds exuded to mobilize inorganic P in soils (Carvalhais et al., 2011; Krishnapriya and Pandey, 2016; Lyu et al., 2016; Giles et al., 2017), solubilizing P bound to iron or aluminum (hydr)oxides (Otani et al., 1996). It was recently discovered that in response to abiotic stress, plant can control malate efflux anions channel (ALMT) which is regulated by anions and gamma-aminobutyric acid (GABA) at the root tip (Ramesh et al., 2015). The anion channels ALMT allow the diffusion of malate along their concentration gradients outside root cells (Gilliham and Tyerman, 2016). In addition, Mora-Macías et al. (2017) found that malate exudation is involved in the signaling of P deficiency. Malate is required for the accumulation of Fe in the apoplast of meristematic cells, triggering the differentiation of meristematic cells in response to Pi deprivation. This activates the root apical meristem exhaustion process, consisting in the loss of meristematic potential and arrest of cell proliferation, ultimately leading to the inhibition of primary root growth and consequently lateral root proliferation. It was also shown that mutants that do not show this response to low P can restore the same P-limitation phenotype by exogenous application of malate in a concentration-dependent manner (Mora-Macías et al., 2017).

Application of exogenous carbohydrates can also initiate the same response (Karthikeyan et al., 2007), albeit with no effect on primary root growth (Hammond and White, 2011). This is interesting as sugars have been shown to be a crucial player in the root response to P-deficiency (Liu et al., 2005; Liu and Vance, 2010; Rouached et al., 2010) and are usually sensed intracellularly (Rolland et al., 2006). The external application implies either that roots can sense external sugar concentrations or that external gradients of organic molecules such as sugars affect internal concentrations either through secondary active uptake or by affecting exudation and efflux rates (by limiting their diffusion to the soil environment). The precise pathways through which sugars can activate the P starvation response is as yet unknown, although several pathways for sugar signaling at the whole plant level have been demonstrated where protein kinases are central in coordinating several of these responses (Hanson and Smeekens, 2009).

When local soil P_i_ concentrations are low, if malate efflux and re-uptake of Fe-malate are necessary to cause the arrest of meristematic activity in primary roots (Mora-Macías et al., 2017), we speculate that it is this response that triggers sugar accumulation (Figure 3). During root apical meristem exhaustion, meristematic cells undergo disruption of symplastic connections (probably due to callose deposition) and cell differentiation (Shishkova et al., 2008; Ogden et al., 2018), changes that would lead to decreased root exudation. Therefore, if sugar transport to the root tip is maintained and meristematic activity is curtailed (decreased loss through root exudation), it would cause a transient increase in sugar concentrations, which represents a potential signal molecule for root architecture. Sugar accumulation is often found in roots in response to P deficiency (Hammond and White, 2011), and the above proposed mechanism would also explain the effect of exogenous applications of sugars on root system architecture. This is mostly related to responses to local P deficiency, while systemic signaling of P homeostasis is likely to include more complex coordinated processes.

### Role of Primary Metabolite Exudation in Nutrient Foraging and Plant-Microbe Interactions

A growing number of studies show that the root tip functions as control center for sensing external nutrient concentrations and translating this into an alteration of root system architecture (Desnos, 2008; Baluška et al., 2010; Péret et al., 2011; Baluška and Mancuso, 2013). The root tip is also the hot spot of root exudation, where amino acids, organic acids, and sugars represent the major chemical classes exuded. Each of these compounds can be sensed in the root by different mechanisms, and all have effects on the root system architecture in a concentration dependent manner, as described above (see Figure 3). In order to increase the cellular concentration in the root tip, changes need to occur either at the source level (phloem loading and transport) or at the sink level (phloem unloading, metabolism, exudation, and microbial consumption). These changes depend on the nutritional status of the plant and the soil environment. Therefore, the flow of root exudates could represent a key determinate of root growth and development, by affecting temporary concentrations of organic solutes at the root tip. Even though we know a great deal about the concentrations of solutes in whole roots, our knowledge of the spatial and temporal dynamics of root organic solutes, especially at the root tip, is highly limited.

More recent studies have appointed the transition zone (Figure 1) as an area acting as the command center for root responses to the environment (Baluška et al., 2010; Baluška and Mancuso, 2013). Baluška and Mancuso (2013) showed that cells in the transition zone are highly active in terms of cytoskeletal rearrangements, endocytosis and endocytic vesicle recycling, as well as in electrical activities. In addition, we previously highlighted that two members of the GLR family are now known to be preferentially expressed in the root tip phloem where they have a role in regulating lateral root initiation (Vincill et al., 2013) and where they may affect root growth *via* electrical signals modulating phloem loading or transport (Baluška and Mancuso, 2013). This indicates that the root exudation process takes place in a highly restricted root area to translate environmental cues into signals for root system development. It is therefore tempting to speculate that any changes in relative concentration gradients of metabolites at the root tip caused by changes of either influx (phloem) and/or efflux (exudates) will signal the transition zone, which could then act as a command center for root growth and architecture.

Soil micro-organisms also add a great complexity to our understanding of the involvement of primary metabolites in plant nutrient foraging. Indeed, micro-organisms quickly consume these compounds, making it practically impossible to collect pure root exudates in real soil environments (Oburger and Jones, 2018). Soil microbes can also affect the root exudation process. We previously highlighted that soil microbes can release secondary compounds to stimulate root amino acid exudation (Phillips et al., 2004), while specific soil microbes were shown to have the ability to modify the metabolite composition of the whole plant (Curzi et al., 2008; Fernandez et al., 2012). This highlights a bi-directional influence between roots and their rhizosphere microbial communities. At the same time, changes in the temporal concentration of primary metabolites at the root tip can affect root system architecture, as discussed above. Therefore, plant strategies for nutrient foraging may be strongly affected by the plant-associated microbial population (Pii et al., 2015a; Alegria Terrazas et al., 2016). Indeed, root-microbe interactions have been shown to affect whole plant growth, particularly *via* effects of rhizosphere microbes on root system architecture and consequent effects on plant aboveground performance (Verbon and Liberman, 2016). In recent years, many studies have discovered plant systemic regulation networks of nutrient sensing that involves cross communications between the below and aboveground part of the plant (Reid et al., 2011a,b; Ohkubo et al., 2017; Tsikou et al., 2018). Therefore, plant-microbe interactions at the root level may exert stronger effects than we think on the whole plant and on nutrient cycling. A recent study demonstrated that root exudation is linked as a functional trait to nutritional strategies (exploitative vs. conservative) of plants (Guyonnet et al., 2018), mostly explained by links to root traits (Guyonnet et al., 2018; Herz et al., 2018). Because a different rhizosphere microbiome develops depending on soil and plant type (Berg and Smalla, 2009), the specific plant-soil feedback can be variable. Yet, it is undeniable that the interplays between plant and soil microbes can shape an ecosystem because of the strong interactions between plant nutritional strategy (and therefore its root exudates) and the soil microbes (Teste et al., 2017).

## Implications at the Ecosystem Scale

### Decomposition of Organic Matter and CO_2_ Emissions

Terrestrial belowground C allocation represents an important process linking plant C fixation to soil C losses, exercising a strong control on soil respiration (Mencuccini and Hölttä, 2010), and structuring the soil microbial community (Eisenhauer et al., 2017), leading to enhanced or reduced soil organic matter decomposition (Kuzyakov, 2010). Many studies have shown that the “autotrophic” component of soil respiration (including roots, associated mycorrhizal fungi, and rhizosphere microbes dependent on recently plant-fixed C) accounts for a significant fraction of soil CO_2_ efflux (Högberg et al., 2001; Scott-Denton et al., 2006; Bahn et al., 2008; Burri et al., 2014). Therefore, understanding how plants exude C and control belowground C fluxes in response to the environment is pivotal. Furthermore, root exudates as an input of labile C substrate can stimulate the decomposition of the more stable soil organic matter pool (Kuzyakov, 2010). This process is termed “rhizosphere priming” and depends among other factors on the amount and chemical composition of labile substrates added to the soils relative to the active microbial biomass (Kuzyakov and Blagodatskaya, 2015). Different organic compounds have been shown to cause different degrees of priming (Hamer and Marschner, 2005), highlighting the complexity of this process. It has recently been proposed that the priming effect responds to the relative availability of N and P, following contrasting results from two experimental grassland studies (Dijkstra et al., 2013). The authors argued that two opposing mechanisms operate when N or P becomes limiting to plants. Soil N is mostly present as high-molecular-weight organic material, while P can be present in both forms, organic and inorganic P. Priming the decomposition of organic matter could therefore be beneficial for plants mining for N (Meier et al., 2017). Instead the release of P can follow different pathways, such as hydrolysis of organic P compounds by extracellular enzymes or Pi desorption/dissolution through enhanced exudation of organic acids, both of which do not necessarily involve carbon release due to SOM decomposition. Our explanation of root exudate responses to nutrient limitation could further support the hypothesis put forward by Dijkstra et al. (2013). Indeed, when P is limiting and meristematic activity stops, this will curtail the exudation of C-rich metabolites (such as sugars) over time that act as a labile source of C to micro-organisms, consequently decreasing the rhizosphere priming effect. On the other hand, under N limiting conditions, low concentrations of organic nitrogen outside the root cells may cause a stronger concentration gradient and increase the flux of exudates to the rhizosphere microbial community, eventually causing an enhanced rhizosphere priming effect. In contrast, roots reaching organic-rich or nutrient-rich patches with high microbial depolymerization activity will encounter enhanced amino acid concentrations, effectively downregulating the efflux of amino acids and eventually other compounds (because of the slowdown of the meristematic activity and therefore regulating sink activity; see Section 3.1) and reducing the rhizosphere priming effect. Therefore, further investigations into the relationship between soil nutrient availability, plant nutrient status, and root exudation will greatly help to predict the consequences for SOC turnover. Indeed, a recent study has shown for the first time that plant nutrient strategy is tightly linked to exudation of primary metabolites, which have significant correlations with soil denitrification (Guyonnet et al., 2017). Specifically, targeted investigations are needed on the effect of biological processes (source-sink activity, efflux carrier expression, etc.) and environmental stresses on the flux of solutes from the roots under realistic conditions, with a great challenge posed by the overwhelming difficulty in collecting representative root exudate samples (Oburger and Jones, 2018).

### Plant Community Composition, Plant Productivity, and Agricultural Production

Plant community composition responds to environmental stresses and to competition for available resources, either aboveground (e.g., light) or belowground (e.g., nutrients and water). Root exudates, nutrient availability, and soil microbial communities form a close interaction network, simultaneously affecting plant growth and competition (Pierik et al., 2013). While many specific compounds in root exudates playing a role in plant community composition have been characterized, such as allelopathic compounds (e.g., sorgoleone in *Sorghum bicolor*) (Bertin et al., 2003), the multitude of compounds exuded by roots poses a challenge in understanding the mechanisms of plant-plant interactions and to decipher those compounds that play a major role. Understanding whether specific root exudates or a combination of them are involved in plant recognition remains a challenge. Therefore, it also remains unresolved whether root exudation of primary metabolites plays an important role in plant-plant interactions as we suggested for nutrient sensing and signaling. A few studies have demonstrated that the concentration of specific root exudates is an important trigger in self-recognition and recognition of other plants (Caffaro et al., 2011). Indeed accumulation of “own” compounds secreted by the same species inhibited root growth (Falik et al., 2005; Semchenko et al., 2007) and played a role in self-recognition and neighbor-recognition (Caffaro et al., 2011, 2013). It was also shown that responses to nutrients can become dominant compared to avoidance of neighboring plants, and that plants integrate signals from both nutrients and competitors (Cahill et al., 2010). Therefore, the ultimate response of plants to their neighbors depends on the complex interplay of mechanisms that integrate numerous environmental cues in which root exudates are undoubtedly involved. Recent studies have shown how plant-soil feedbacks, mostly determined by the relationship between the plant and the soil microbial community, can determine plant performance and community structure (Kardol et al., 2007; Reinhart, 2012; Ma et al., 2017; Van Nuland et al., 2017; Bukowski et al., 2018; Eppinga et al., 2018; Hu et al., 2018; Wubs and Bezemer, 2018). Indeed, in recent experiments, it was shown that effects on plant performance by soil microbiota through plant-soil feedbacks were dependent on the plant nutrient strategy and soil nutrient conditions (Png et al., 2019; Teste et al., 2017). More specifically, Teste et al. (2017) revealed that these nutritional strategy-dependent feedbacks were the key in maintaining plant functional and taxonomic diversity in Mediterranean-climate shrublands. These results are extremely important and highlight our need to understand how plant nutrient strategies affect root exudation patterns and how this can elicit feedback on plant community assembly.

Understanding the role of root exudates in plant recognition and competition is likely to have significant impacts on agricultural productivity. Modern agriculture calls for sustainable systems that can maintain productivity while reducing inputs and losses and increasing the biodiversity of agro-ecosystems (Foley et al., 2011). Highly successful results were obtained with intercropping systems, where two or more crop species were grown together to maximize light and resource use while reducing susceptibility to pests, an approach that could represent a highly valuable tool for increasing the sustainability of food production (Brooker et al., 2015; Mariotte et al., 2018). In this context, understanding belowground interactions determining competition and facilitation and nutrient foraging strategies is essential (Giles et al., 2017). For example, it was shown that root exudates of maize promoted N_2_ fixation of faba bean, whereas root exudates of barley and wheat did not (Li et al., 2016). Indeed, in agro-ecosystems, the natural complexity can be reduced, as plant community composition, nutrient, and water regimes can all be manipulated. By reducing the complexity of plant-plant and plant-microbe interactions, it might become easier to successfully apply fundamental research to agricultural systems. However, more studies need to specifically address the role of root exudates in nutrient foraging and plant recognition in order to understand the mechanisms involved and successfully develop combinations of plant species and soil microbes that improve the sustainability of food production.

## Summary

In this manuscript, we synthesized recent advances in biology to explain and propose a mechanism by which root exudation of primary metabolites is controlled, and how this process can affect plant nutrient acquisition strategies and root system architecture. We propose two concepts to build a mechanistic understanding of how root exudation can affect plant nutrition:

1. *Root exudation of primary metabolites is driven by facilitated diffusion but plants and microbes can control the root exudation process by modifying concentration gradients depending on their nutritional status*:
  Root exudation occurs mainly at the root meristematic apex, where fluxes of metabolites into the soil are the highest, due to the lack of root endodermis differentiation. Concentration gradients are fundamental in driving root exudation as most of the primary metabolites efflux from root cells through membrane transporters that facilitate diffusion. Soil microbes actively consume root exudates and keep concentrations of the respective substance low in the soil environment and at the same time can stimulate specific root exudation patterns. Plants, on the other side, can control concentration gradients by different mechanisms: they can adjust the supply rate through phloem loading and transport (source activity) or modify phloem unloading and sink activity, including the inhibition of root meristematic activity or up/downregulation of efflux transporters. The functioning of efflux carriers can also be controlled by rapid post-translational mechanisms (e.g., phosphorylation), which suggest that a fast control of root exudation is possible. The exudation flux ultimately will reflect the nutritional status and strategy of the plant or of the micro-organisms (soil environment). While plant source-sink dynamics are increasingly recognized as being fundamental to understanding C allocation and nutrient uptake, we support the idea that plant-associated micro-organisms affect plant carbon allocation by affecting concentration gradients that drive root exudation (Farrar and Jones, 2000; Savage et al., 2016). Hence, we introduce a novel conceptual framework for the control of root exudation that considers source-sink dynamics of plants and micro-organisms together as an integrated system.
2. *Changes in the concentration of primary metabolites at the root tip are sensed by the plant and signals are translated to modify root system architecture*:
  We present multiple examples of how important root metabolite concentrations can be increased or decreased inside the root tip. Specifically, regulation of efflux transporter expression in roots, root exudate re-uptake, phloem loading, and modulation of meristematic activity are major mechanisms that integrate the root exudation process with the soil environment surrounding the root tips. Modulating this flux can change the temporal concentrations of solutes inside the root tip, which elicits responses in root system architecture to modulate root growth to environmental cues. Indeed, the three chemical groups mentioned above (sugars, amino acids and organic acids), comprising a vast proportion of the root exudates, can all be sensed in different ways and induce a range of responses in the root system architecture from lateral root formation to root meristem exhaustion, depending on the plant and soil nutritional status. Soil microbes can create specific patterns of metabolite concentrations around the root tip by preferentially consuming some metabolites and by stimulating plant exudation. Therefore, soil microbes can exert a strong control on plant nutrient sensing.

**Figure 4:**
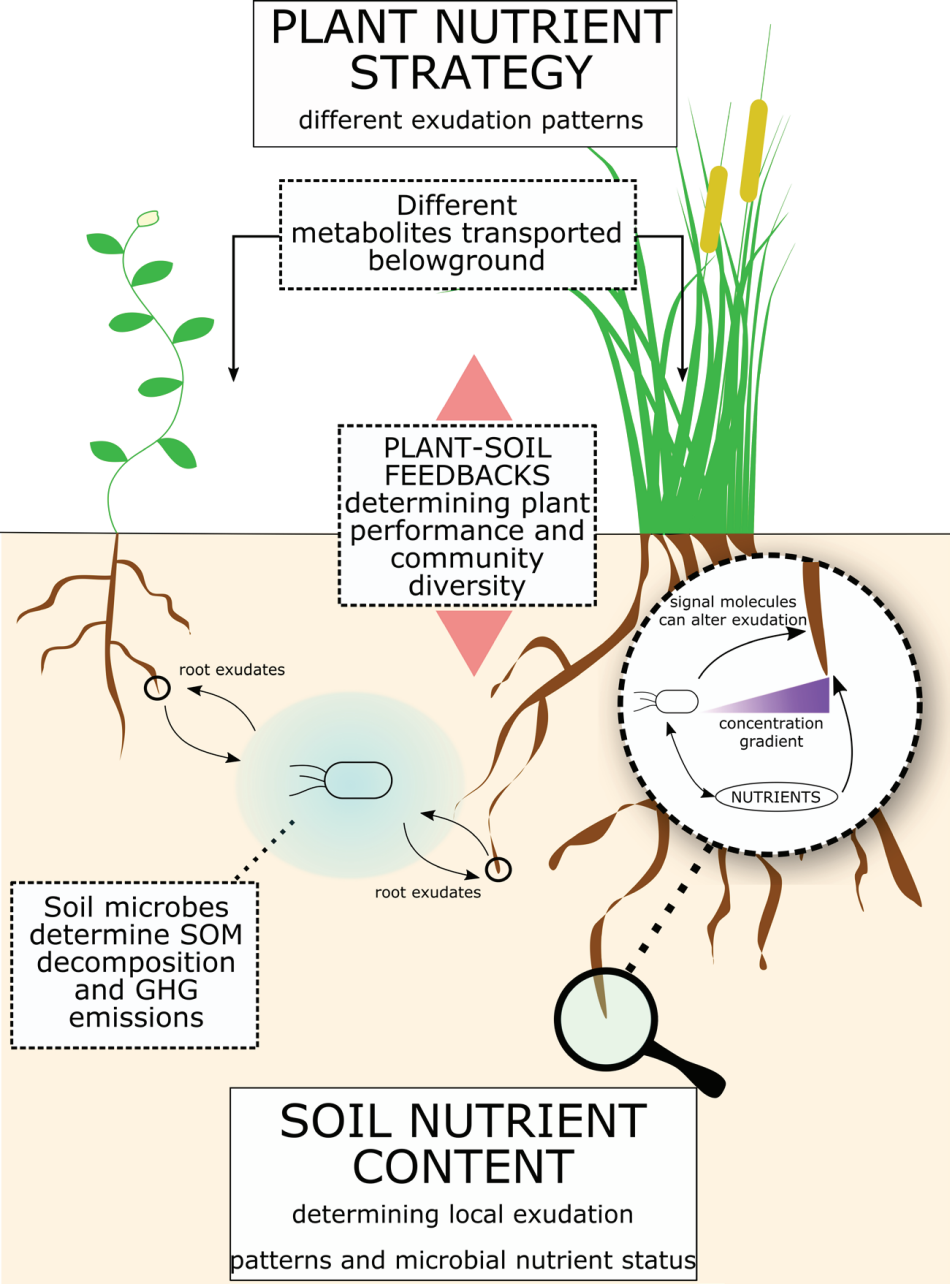
Root exudates link soil nutrients to plant nutrient strategy. Figure illustrates a summary of the concepts proposed for the role of root exudates in plant-microbe interactions and consequences for ecosystems. Plant nutrient strategy determines different exudation patterns, while the soil physical and nutrient composition determines the microbial community and its nutrient demand. Root exudation is a flux with a bi-directional influence between plant and soil microbes. Indeed, while plants can regulate root exudation, soil micro-organism can enhance exudation rates and release molecules that affect root exudation patterns. Ultimately, root exudation has a significant effect on SOM decomposition and greenhouse gases (GHGs) release from soil. Also, plant-soil feedbacks can significantly affect plant performance and plant community composition.

This proposed framework has strong repercussions on the understanding of ecosystem functioning (Figure 4). Indeed, plant belowground C allocation and root exudation in response to environmental changes has attracted much interest, especially on the implications for soil C sequestration and losses. Yet, a conclusive mechanistic explanation of the underlying processes has remained elusive. In comparison to fertilization, little research has targeted the manipulation of plant carbon transport and sensing of organic molecules for improving agricultural productivity (Tegeder and Masclaux-Daubresse, 2018), even though a decrease in fertilization and an increase in agricultural sustainability is called for. Studies on plant-microbe interactions are currently increasing, and it will be pivotal to understand the links between primary metabolite exudation and soil micro-organisms. Indeed, exudation of primary metabolites has strong effects on SOM decomposition by soil microbes, and primary metabolite exudation patterns are linked to plant nutrient strategies, which through plant-soil feedback mechanisms can determine an ecosystem performance. Also how mycorrhizal association affects root exudation and the processes influenced thereby is still largely unknown, largely due to having a majority of studies being performed with the non-mycorrhizal plant *Arabidopsis*, leaving uncertainties on their potential use in agriculture (Thirkell et al., 2017). Despite the undeniable importance of nutrient uptake transporters, we here suggest that exudation of primary metabolites and plant-microbe interactions plays a pivotal role in nutrient uptake and plant community dynamics and that their mechanistic understanding will be crucial in building more nutrient-efficient agro-ecosystems.

## Author Contributions

AC conceived and designed the manuscript structure. AC, WW, and CK collected references and wrote the manuscript with substantial contributions from AM and AR.

### Conflict of Interest Statement

The authors declare that the research was conducted in the absence of any commercial or financial relationships that could be construed as a potential conflict of interest.

## References

[ref1] Ahmed M. A. Passioura J. Carminati A. (2018). Hydraulic processes in roots and the rhizosphere pertinent to increasing yield of water-limited grain crops: a critical review. J. Exp. Bot. 69, 3255–3265. 10.1093/jxb/ery183, PMID: 29767797

[ref2] Alegria Terrazas R. Giles C. Paterson E. Robertson-Albertyn S. Cesco S. Mimmo T. (2016). “Chapter one: Plant–microbiota interactions as a driver of the mineral turnover in the rhizosphere,” in Advances in applied microbiology. eds. Sariaslani S. Gadd G. M. (Cambridge, Massachusetts, USA: Academic Press), 1–67. 10.1016/bs.aambs.2016.03.001 27261781

[ref3] Aslam M. Travis R. L. Rains D. W. (2001). Differential effect of amino acids on nitrate uptake and reduction systems in barley roots. Plant Sci. 160, 219–228. 10.1016/S0168-9452(00)00391-5, PMID: 11164593

[ref4] Badri D. V. Loyola-Vargas V. M. Du J. Stermitz F. R. Broeckling C. D. Iglesias-Andreu L.. (2008). Transcriptome analysis of Arabidopsis roots treated with signaling compounds: a focus on signal transduction, metabolic regulation and secretion. New Phytol. 179, 209–223. 10.1111/j.1469-8137.2008.02458.x, PMID: 18422893

[ref5] Badri D. V. Quintana N. El Kassis E. G. Kim H. K. Choi Y. H. Sugiyama A.. (2009). An ABC transporter mutation alters root exudation of phytochemicals that provoke an overhaul of natural soil microbiota. Plant Physiol. 151, 2006–2017. 10.1104/pp.109.147462, PMID: 19854857 PMC2785968

[ref6] Badri D. V. Vivanco J. M. (2009). Regulation and function of root exudates. Plant Cell Environ. 32, 666–681. 10.1111/j.1365-3040.2009.01926.x 19143988

[ref7] Bahn M. Rodeghiero M. Anderson-Dunn M. Dore S. Gimeno C. Drösler M.. (2008). Soil respiration in European grasslands in relation to climate and assimilate supply. Ecosystems 11, 1352–1367. 10.1007/s10021-008-9198-0, PMID: 20936099 PMC2950939

[ref8] Baluška F. Mancuso S. (2013). Root apex transition zone as oscillatory zone. Front. Plant Sci. 4, 354. 10.3389/fpls.2013.00354, PMID: 24106493 PMC3788588

[ref9] Baluška F. Mancuso S. Volkmann D. Barlow P. W. (2010). Root apex transition zone: a signalling–response nexus in the root. Trends Plant Sci. 15, 402–408. 10.1016/j.tplants.2010.04.007, PMID: 20621671

[ref10] Berg G. Smalla K. (2009). Plant species and soil type cooperatively shape the structure and function of microbial communities in the rhizosphere. FEMS Microbiol. Ecol. 68, 1–13. 10.1111/j.1574-6941.2009.00654.x 19243436

[ref11] Bertin C. Yang X. Weston L. A. (2003). The role of root exudates and allelochemicals in the rhizosphere. Plant Soil 256, 67–83. 10.1023/A:1026290508166

[ref12] Besnard J. Pratelli R. Zhao C. Sonawala U. Collakova E. Pilot G.. (2016). UMAMIT14 is an amino acid exporter involved in phloem unloading in Arabidopsis roots. J. Exp. Bot. 67, 6385–6397. 10.1093/jxb/erw412, PMID: 27856708 PMC5181585

[ref13] Bharadwaj D. P. Alström S. Lundquist P.-O. (2012). Interactions among *Glomus irregulare*, arbuscular mycorrhizal spore-associated bacteria, and plant pathogens under in vitro conditions. Mycorrhiza 22, 437–447. 10.1007/s00572-011-0418-7, PMID: 22081167

[ref14] Biondini M. Klein d. A. Redente e. F. (1988). Carbon and nitrogen losses through root exudation by *Agropyron cristatum*, *A smithii* and *Bouteloua gracilis*. Soil Biol. Biochem. 20, 477–482. 10.1016/0038-0717(88)90061-2

[ref15] Bonfante P. Requena N. (2011). Dating in the dark: how roots respond to fungal signals to establish arbuscular mycorrhizal symbiosis. Curr. Opin. Plant Biol. 14, 451–457. 10.1016/j.pbi.2011.03.014, PMID: 21489861

[ref16] Brooker R. W. Bennett A. E. Cong W. -F. Daniell T. J. George T. S. Hallett P. D.. (2015). Improving intercropping: a synthesis of research in agronomy, plant physiology and ecology. New Phytol. 206, 107–117. 10.1111/nph.13132, PMID: 25866856

[ref17] Brundrett M. C. (2009). Mycorrhizal associations and other means of nutrition of vascular plants: understanding the global diversity of host plants by resolving conflicting information and developing reliable means of diagnosis. Plant Soil 320, 37–77. 10.1007/s11104-008-9877-9

[ref18] Bukowski A. R. Schittko C. Petermann J. S. (2018). The strength of negative plant-soil feedback increases from the intraspecific to the interspecific and the functional group level. Ecol. Evol. 8, 2280–2289. 10.1002/ece3.3755, PMID: 29468043 PMC5817124

[ref19] Burri S. Sturm P. Prechsl U. E. Knohl A. Buchmann N. (2014). The impact of extreme summer drought on the short-term carbon coupling of photosynthesis to soil CO_2_ efflux in a temperate grassland. Biogeosciences 11, 961–975. 10.5194/bg-11-961-2014

[ref20] Caffaro M. M. Vivanco J. M. Boem F. H. G. Rubio G. (2011). The effect of root exudates on root architecture in *Arabidopsis thaliana*. Plant Growth Regul. 64, 241–249. 10.1007/s10725-011-9564-3

[ref21] Caffaro M. M. Vivanco J. M. Botto J. Rubio G. (2013). Root architecture of *Arabidopsis* is affected by competition with neighbouring plants. Plant Growth Regul. 70, 141–147. 10.1007/s10725-013-9786-7

[ref22] Cahill J. F. McNickle G. G. Haag J. J. Lamb E. G. Nyanumba S. M. St. Clair C. C. (2010). Plants integrate information about nutrients and neighbors. Science 328, 1657–1657. 10.1126/science.1189736, PMID: 20576883

[ref23] Caputo C. Barneix A. J. (1997). Export of amino acids to the phloem in relation to N supply in wheat. Physiol. Plant. 101, 853–860. 10.1111/j.1399-3054.1997.tb01073.x

[ref24] Carvalhais L. C. Dennis P. G. Fedoseyenko D. Hajirezaei M. R. Borriss R. von Wiren N. (2011). Root exudation of sugars, amino acids, and organic acids by maize as affected by nitrogen, phosphorus, potassium, and iron deficiency. J. Plant Nutr. Soil Sci. 174, 3–11. 10.1002/jpln.201000085

[ref25] Chen H. -Y. Huh J. -H. Yu Y. -C. Ho L. -H. Chen L. -Q. Tholl D.. (2015). The Arabidopsis vacuolar sugar transporter SWEET2 limits carbon sequestration from roots and restricts Pythium infection. Plant J. 83, 1046–1058. 10.1111/tpj.12948, PMID: 26234706

[ref26] Cheng L. Booker F. L. Tu C. Burkey K. O. Zhou L. Shew H. D.. (2012). Arbuscular mycorrhizal fungi increase organic carbon decomposition under elevated CO_2_. Science 337, 1084–1087. 10.1126/science.1224304, PMID: 22936776

[ref27] Cheng L. Zhu J. Chen G. Zheng X. Oh N. -H. Rufty T. W.. (2010). Atmospheric CO_2_ enrichment facilitates cation release from soil. Ecol. Lett. 13, 284–291. 10.1111/j.1461-0248.2009.01421.x, PMID: 20100242

[ref28] Chevalier F. Pata M. Nacry P. Doumas P. Rossignol M. (2003). Effects of phosphate availability on the root system architecture: large-scale analysis of the natural variation between Arabidopsis accessions. Plant Cell Environ. 26, 1839–1850. 10.1046/j.1365-3040.2003.01100.x

[ref29] Chiu J. C. Brenner E. D. DeSalle R. Nitabach M. N. Holmes T. C. Coruzzi G. M. (2002). Phylogenetic and expression analysis of the glutamate-receptor-like gene family in *Arabidopsis thaliana*. Mol. Biol. Evol. 19, 1066–1082. 10.1093/oxfordjournals.molbev.a004165, PMID: 12082126

[ref30] Curzi M. J. Ribaudo C. M. Trinchero G. D. Curá J. A. Pagano E. A. (2008). Changes in the content of organic and amino acids and ethylene production of rice plants in response to the inoculation with *Herbaspirillum seropedicae*. J. Plant Interact. 3, 163–173. 10.1080/17429140802255167

[ref31] Dakora F. D. Phillips D. A. (2002). Root exudates as mediators of mineral acquisition in low-nutrient environments. Plant Soil 245, 35–47. 10.1023/a:1020809400075

[ref32] Darwent M. J. Paterson E. McDonald A. J. S. Tomos A. D. (2003). Biosensor reporting of root exudation from *Hordeum vulgare* in relation to shoot nitrate concentration. J. Exp. Bot. 54, 325–334. 10.1093/jxb/erg017, PMID: 12493860

[ref33] De Schepper V. De Swaef T. Bauweraerts I. Steppe K. (2013). Phloem transport: a review of mechanisms and controls. J. Exp. Bot. 64, 4839–4850. 10.1093/jxb/ert302, PMID: 24106290

[ref34] Dennison K. L. Spalding E. P. (2000). Glutamate-gated calcium fluxes in Arabidopsis. Plant Physiol. 124, 1511–1514. 10.1104/pp.124.4.1511, PMID: 11115867 PMC1539305

[ref35] Depuydt S. (2014). Arguments for and against self and non-self root recognition in plants. Front. Plant Sci. 5:614 10.3389/fpls.2014.00614 25414719 PMC4222137

[ref36] Desnos T. (2008). Root branching responses to phosphate and nitrate. Curr. Opin. Plant Biol. 11, 82–87. 10.1016/j.pbi.2007.10.003, PMID: 18024148

[ref37] Dijkstra F. A. Carrillo Y. Pendall E. Morgan J. A. (2013). Rhizosphere priming: a nutrient perspective. Front. Microbiol. 4:216. 10.3389/fmicb.2013.00216, PMID: 23908649 PMC3725428

[ref38] Dinkeloo K. Boyd S. Pilot G. (2017). Update on amino acid transporter functions and on possible amino acid sensing mechanisms in plants. Semin. Cell Dev. Biol. 74, 105–113. 10.1016/j.semcdb.2017.07.010 28705659

[ref39] Dluzniewska P. Gessler A. Kopriva S. Strnad M. NovÁK O. Dietrich H.. (2006). Exogenous supply of glutamine and active cytokinin to the roots reduces NO_3_–uptake rates in poplar. Plant Cell Environ. 29, 1284–1297. 10.1111/j.1365-3040.2006.01507.x, PMID: 17080950

[ref40] Doan T. H. Doan T. A. Kangas M. J. Ernest A. E. Tran D. Wilson C. L. (2017). A low-cost imaging method for the temporal and spatial colorimetric detection of free amines on maize root surfaces. Front. Plant Sci. 8:1513. 10.3389/fpls.2017.01513 28912796 PMC5582365

[ref41] Domínguez-May Á. V. Carrillo-Pech M. Barredo-Pool F. A. Martínez-Estévez M. Us-Camas R. Y. Moreno-Valenzuela O. A. (2013). A novel effect for glycine on root system growth of habanero pepper. J. Am. Soc. Hortic. Sci. 138, 433–442. 10.21273/JASHS.138.6.433

[ref42] Drigo B. Pijl A. S. Duyts H. Kielak A. M. Gamper H. A. Houtekamer M. J.. (2010). Shifting carbon flow from roots into associated microbial communities in response to elevated atmospheric CO_2_. Proc. Natl. Acad. Sci. 107, 10938–10942. 10.1073/pnas.0912421107, PMID: 20534474 PMC2890735

[ref43] Eisenhauer N. Lanoue A. Strecker T. Scheu S. Steinauer K. Thakur M. P.. (2017). Root biomass and exudates link plant diversity with soil bacterial and fungal biomass. Sci. Rep. 7:44641 10.1038/srep44641, PMID: 28374800 PMC5379681

[ref44] el Zahar Haichar F. Santaella C. Heulin T. Achouak W. (2014). Root exudates mediated interactions belowground. Soil Biol. Biochem. 77, 69–80. 10.1016/j.soilbio.2014.06.017

[ref45] Eppinga M. B. Baudena M. Johnson D. J. Jiang J. Mack K. M. L. Strand A. E. (2018). Frequency-dependent feedback constrains plant community coexistence. Nat. Ecol. Evol. 2, 1403–1407. 10.1038/s41559-018-0622-3 30061563

[ref46] Evans M. L. Ishikawa H. Estelle M. A. (1994). Responses of Arabidopsis roots to auxin studied with high temporal resolution: comparison of wild type and auxin-response mutants. Planta 194, 215–222. 10.1007/bf00196390

[ref47] Falik O. Reides P. Gersani M. Novoplansky A. (2005). Root navigation by self inhibition. Plant Cell Environ. 28, 562–569. 10.1111/j.1365-3040.2005.01304.x

[ref48] Farrar J. F. Jones D. L. (2000). The control of carbon acquisition by roots. New Phytol. 147, 43–53. 10.1046/j.1469-8137.2000.00688.x

[ref49] Fatichi S. Leuzinger S. Körner C. (2014). Moving beyond photosynthesis: from carbon source to sink-driven vegetation modeling. New Phytol. 201, 1086–1095. 10.1111/nph.12614, PMID: 24261587

[ref50] Fernandez O. Theocharis A. Bordiec S. Feil R. Jacquens L. Clément C.. (2012). Burkholderia phytofirmans PsJN acclimates grapevine to cold by modulating carbohydrate metabolism. Mol. Plant-Microbe Interact. 25, 496–504. 10.1094/MPMI-09-11-0245, PMID: 22409157

[ref51] Fischer W.-N. Loo D. D. F. Koch W. Ludewig U. Boorer K. J. Tegeder M.. (2002). Low and high affinity amino acid H+−cotransporters for cellular import of neutral and charged amino acids. Plant J. 29, 717–731. 10.1046/j.1365-313X.2002.01248.x, PMID: 12148530

[ref52] Foley J. A. Ramankutty N. Brauman K. A. Cassidy E. S. Gerber J. S. Johnston M.. (2011). Solutions for a cultivated planet. Nature 478, 337–342. 10.1038/nature10452, PMID: 21993620

[ref53] Forde B. G. (2014). Nitrogen signalling pathways shaping root system architecture: an update. Current opinion in plant biology 21, 30–36. PMID: 24997289 10.1016/j.pbi.2014.06.004

[ref54] Forde B. G. Lea P. J. (2007). Glutamate in plants: metabolism, regulation, and signalling. J. Exp. Bot. 58, 2339–2358. 10.1093/jxb/erm121, PMID: 17578865

[ref55] Forsum O. (2016). On plant responses to D-amino acids. (Doctoral dissertation). Retrieved from: https://pub.epsilon.slu.se/12997/

[ref56] Forsum O. Svennerstam H. Ganeteg U. Näsholm T. (2008). Capacities and constraints of amino acid utilization in Arabidopsis. New Phytol. 179, 1058–1069. 10.1111/j.1469-8137.2008.02546.x, PMID: 18627491

[ref57] Friedel J. K. Scheller E. (2002). Composition of hydrolysable amino acids in soil organic matter and soil microbial biomass. Soil Biol. Biochem. 34, 315–325. 10.1016/S0038-0717(01)00185-7, PMID: 12494383

[ref58] Fusconi A. (2014). Regulation of root morphogenesis in arbuscular mycorrhizae: what role do fungal exudates, phosphate, sugars and hormones play in lateral root formation? Ann. Bot. 113, 19–33. 10.1093/aob/mct258, PMID: 24227446 PMC3864729

[ref59] Gent L. Forde B. G. (2017). How do plants sense their nitrogen status? J. Exp. Bot. 68, 2531–2539. 10.1093/jxb/erx013, PMID: 28201547

[ref60] Giehl R. F. H. von Wirén N. (2014). Root nutrient foraging. Plant Physiol. 166, 509–517. 10.1104/pp.114.245225, PMID: 25082891 PMC4213083

[ref61] Giles C. D. Brown L. K. Adu M. O. Mezeli M. M. Sandral G. A. Simpson R. J.. (2017). Response-based selection of barley cultivars and legume species for complementarity: root morphology and exudation in relation to nutrient source. Plant Sci. 255, 12–28. 10.1016/j.plantsci.2016.11.002, PMID: 28131338

[ref62] Gill A. L. Finzi A. C. (2016). Belowground carbon flux links biogeochemical cycles and resource-use efficiency at the global scale. Ecol. Lett. 19, 1419–1428. 10.1111/ele.12690, PMID: 27758027

[ref63] Gilliham M. Tyerman S. D. (2016). Linking metabolism to membrane signaling: the GABA-Malate connection. Trends Plant Sci. 21, 295–301. 10.1016/j.tplants.2015.11.011, PMID: 26723562

[ref64] Gioseffi E. de Neergaard A. Schjoerring J. K. (2012). Interactions between uptake of amino acids and inorganic nitrogen in wheat plants. Biogeosciences 9, 1509–1518. 10.5194/bg-9-1509-2012

[ref65] Godt D. Roitsch T. (2006). The developmental and organ specific expression of sucrose cleaving enzymes in sugar beet suggests a transition between apoplasmic and symplasmic phloem unloading in the tap roots. Plant Physiol. Biochem. 44, 656-665. 10.1016/j.plaphy.2006.09.019, PMID: 17095237

[ref218] Gorka S. Dietrich M. Mayerhofer W. Gabriel R. Wiesenbauer J. Martin V. Rapid transfer of plant photosynthates to soil bacteria via ectomycorrhizal hyphae and its interaction with nitrogen availability. Front. Microbiol. 10. 10.3389/fmicb.2019.00168 30863368 PMC6399413

[ref66] Grimoldi A. A. Kavanová M. Lattanzi F. A. Schäufele R. Schnyder H. (2006). Arbuscular mycorrhizal colonization on carbon economy in perennial ryegrass: quantification by 13CO_2_/12CO_2_ steady-state labelling and gas exchange. New Phytol. 172, 544–553. 10.1111/j.1469-8137.2006.01853.x, PMID: 17083684

[ref67] Groleau-Renaud V. Plantureux S. Tubeileh A. Guckert A. (2000). Influence of microflora and composition of root bathing solution on root exudation of maize plants. J. Plant Nutr. 23, 1283–1301. 10.1080/01904160009382100

[ref68] Gutjahr C. Parniske M. (2013). Cell and developmental biology of arbuscular mycorrhiza symbiosis. Annu. Rev. Cell Dev. Biol. 29, 593–617. 10.1146/annurev-cellbio-101512-122413, PMID: 24099088

[ref69] Guyonnet J. P. Cantarel A. A. M. Simon L. Haichar F. e. Z. (2018). Root exudation rate as functional trait involved in plant nutrient-use strategy classification. Ecol. Evol. 8, 8573–8581. 10.1002/ece3.4383, PMID: 30250724 PMC6144958

[ref70] Guyonnet J. P. Vautrin F. Meiffren G. Labois C. Cantarel A. A. M. Michalet S.. (2017). The effects of plant nutritional strategy on soil microbial denitrification activity through rhizosphere primary metabolites. FEMS Microbiol. Ecol. 93:fix022 10.1093/femsec/fix022, PMID: 28334144

[ref71] Hamer U. Marschner B. (2005). Priming effects in different soil types induced by fructose, alanine, oxalic acid and catechol additions. Soil Biol. Biochem. 37, 445–454. 10.1016/j.soilbio.2004.07.037, PMID: 16491685

[ref72] Hammond J. P. White P. J. (2011). Sugar signaling in root responses to low phosphorus availability. Plant Physiol. 156, 1033–1040. 10.1104/pp.111.175380, PMID: 21487049 PMC3135921

[ref73] Hanson J. Smeekens S. (2009). Sugar perception and signaling—an update. Curr. Opin. Plant Biol. 12, 562–567. 10.1016/j.pbi.2009.07.014, PMID: 19716759

[ref74] Häusler R. E. Ludewig F. Krueger S. (2014). Amino acids – a life between metabolism and signaling Plant Sci. 229, 225–237. 10.1016/j.plantsci.2014.09.011, PMID: 25443849

[ref75] Hawes M. Allen C. Turgeon B. G. Curlango-Rivera G. Minh Tran T. Huskey D. A.. (2016). Root border cells and their role in plant defense. Annu. Rev. Phytopathol. 54, 143–161. 10.1146/annurev-phyto-080615-100140, PMID: 27215971

[ref76] Hawes M. C. Bengough G. Cassab G. Ponce G. (2002). Root caps and rhizosphere. J. Plant Growth Regul. 21, 352–367. 10.1007/s00344-002-0035-y

[ref77] Henry H. A. L. Jefferies R. L. (2003). Interactions in the uptake of amino acids, ammonium and nitrate ions in the Arctic salt-marsh grass, *Puccinellia phryganodes*. Plant Cell Environ. 26, 419–428. 10.1046/j.1365-3040.2003.00973.x

[ref78] Herz K. Dietz S. Gorzolka K. Haider S. Jandt U. Scheel D.. (2018). Linking root exudates to functional plant traits. PLoS One 13:e0204128. 10.1371/journal.pone.0204128, PMID: 30281675 PMC6169879

[ref79] Hirner A. Ladwig F. Stransky H. Okumoto S. Keinath M. Harms A.. (2006). Arabidopsis LHT1 is a high-affinity transporter for cellular amino acid uptake in both root epidermis and leaf mesophyll. Plant Cell 18, 1931–1946. 10.1105/tpc.106.041012, PMID: 16816136 PMC1533986

[ref80] Högberg P. Nordgren A. Buchmann N. Taylor A. F. S. Ekblad A. Högberg M. N.. (2001). Large-scale forest girdling shows that current photosynthesis drives soil respiration. Nature 411, 789. 10.1038/35081058, PMID: 11459055

[ref81] Hu L. Robert C. A. M. Cadot S. Zhang X. Ye M. Li B. (2018). Root exudate metabolites drive plant-soil feedbacks on growth and defense by shaping the rhizosphere microbiota. Nat. Commun. 9:2738. 10.1038/s41467-018-05122-7 30013066 PMC6048113

[ref82] Inselsbacher E. Näsholm T. (2012). The below-ground perspective of forest plants: soil provides mainly organic nitrogen for plants and mycorrhizal fungi. New Phytol. 195, 329–334. 10.1111/j.1469-8137.2012.04169.x, PMID: 22564239

[ref83] Jaeger C. H. Lindow S. E. Miller W. Clark E. Firestone M. K. (1999). Mapping of sugar and amino acid availability in soil around roots with bacterial sensors of sucrose and tryptophan. Appl. Environ. Microbiol. 65, 2685–2690. PMID: 10347061 10.1128/aem.65.6.2685-2690.1999 PMC91396

[ref84] Jämtgård S. Näsholm T. Huss-Danell K. (2008). Characteristics of amino acid uptake in barley. Plant Soil 302, 221–231. 10.1007/s11104-007-9473-4

[ref85] Jansa J. Bukovská P. Gryndler M. (2013). Mycorrhizal hyphae as ecological niche for highly specialized hypersymbionts or just soil free-riders? Front. Plant Sci. 4:134 10.3389/fpls.2013.00134 23720665 PMC3655320

[ref86] Johnson N. C. Wilson G. W. T. Wilson J. A. Miller R. M. Bowker M. A. (2015). Mycorrhizal phenotypes and the law of the minimum. New Phytol. 205, 1473–1484. 10.1111/nph.13172, PMID: 25417818

[ref87] Jones D. Darrah P. (1993). Re-sorption of organic compounds by roots of Zea mays L. and its consequences in the rhizosphere. Plant Soil 153, 47–59. 10.1007/BF00010543

[ref88] Jones D. L. Nguyen C. Finlay R. D. (2009). Carbon flow in the rhizosphere: carbon trading at the soil-root interface. Plant Soil 321, 5–33. 10.1007/s11104-009-9925-0

[ref89] Jones D. L. Shannon D. Junvee-Fortune T. Farrar J. F. (2005). Plant capture of free amino acids is maximized under high soil amino acid concentrations. Soil Biol. Biochem. 37, 179–181. 10.1016/j.soilbio.2004.07.021

[ref90] Kaiser C. Kilburn M. R. Clode P. L. Fuchslueger L. Koranda M. Cliff J. B.. (2015). Exploring the transfer of recent plant photosynthates to soil microbes: mycorrhizal pathway vs direct root exudation. New Phytol. 205, 1537–1551. 10.1111/nph.13138, PMID: 25382456 PMC4357392

[ref91] Kan C.-C. Chung T.-Y. Wu H.-Y. Juo Y.-A. Hsieh M.-H. (2017). Exogenous glutamate rapidly induces the expression of genes involved in metabolism and defense responses in rice roots. BMC Genomics 18:186. 10.1186/s12864-017-3588-7 28212609 PMC5316172

[ref92] Kanno S. Arrighi J.-F. Chiarenza S. Bayle V. Berthomé R. Péret B.. (2016). A novel role for the root cap in phosphate uptake and homeostasis. elife 5:e14577. 10.7554/eLife.14577, PMID: 27050616 PMC4829427

[ref93] Kardol P. Cornips N. J. van Kempen M. M. L. Bakx-Schotman J. M. T. van der Putten W. H. (2007). Microbe-mediated plant-soil feedback causes historical contingency effects in plant community assembly. Ecol. Monogr. 77, 147–162. 10.1890/06-0502

[ref94] Karthikeyan A. S. Varadarajan D. K. Jain A. Held M. A. Carpita N. C. Raghothama K. G. (2007). Phosphate starvation responses are mediated by sugar signaling in Arabidopsis. Planta 225, 907–918. 10.1007/s00425-006-0408-8, PMID: 17033812

[ref95] Kellermeier F. Armengaud P. Seditas T. J. Danku J. Salt D. E. Amtmann A. (2014). Analysis of the root system architecture of Arabidopsis provides a quantitative readout of crosstalk between nutritional signals. Plant Cell 26, 1480–1496. 10.1105/tpc.113.122101, PMID: 24692421 PMC4036566

[ref96] Khan M. A. Gemenet D. C. Villordon A. (2016). Root system architecture and abiotic stress tolerance: current knowledge in root and tuber crops. Front. Plant Sci. 7:1584. 10.3389/fpls.2016.01584, PMID: 27847508 PMC5088196

[ref97] Knoblauch M. Knoblauch J. Mullendore D. L. Savage J. A. Babst B. A. Beecher S. D.. (2016). Testing the Münch hypothesis of long distance phloem transport in plants. elife 5:e15341. 10.7554/eLife.15341, PMID: 27253062 PMC4946904

[ref98] Kochian L. V. Pineros M. A. Hoekenga O. A. (2005). “The physiology, genetics and molecular biology of plant aluminum resistance and toxicity” in Root physiology: from gene to function. (New York City, USA: Springer), 175–195.

[ref99] Körner C. (2015). Paradigm shift in plant growth control. Curr. Opin. Plant Biol. 25, 107–114. 10.1016/j.pbi.2015.05.003 26037389

[ref100] Kramer E. M. Frazer N. L. Baskin T. I. (2007). Measurement of diffusion within the cell wall in living roots of *Arabidopsis thaliana*. J. Exp. Bot. 58, 3005–3015. 10.1093/jxb/erm155, PMID: 17728296

[ref101] Krishnapriya V. Pandey R. (2016). Root exudation index: screening organic acid exudation and phosphorus acquisition efficiency in soybean genotypes. Crop Pasture Sci. 67, 1096–1109. 10.1071/cp15329

[ref102] Kuzyakov Y. (2010). Priming effects: interactions between living and dead organic matter. Soil Biol. Biochem. 42, 1363–1371. 10.1016/j.soilbio.2010.04.003

[ref103] Kuzyakov Y. Blagodatskaya E. (2015). Microbial hotspots and hot moments in soil: concept and review. Soil Biol. Biochem. 83, 184–199. 10.1016/j.soilbio.2015.01.025

[ref104] Kuzyakov Y. Raskatov A. Kaupenjohann M. (2003). Turnover and distribution of root exudates of Zea mays. Plant Soil 254, 317–327. 10.1023/a:1025515708093

[ref105] Lambers H. Shane M. W. Cramer M. D. Pearse S. J. Veneklaas E. J. (2006). Root structure and functioning for efficient acquisition of phosphorus: matching morphological and physiological traits. Ann. Bot. 98, 693–713. 10.1093/aob/mcl114, PMID: 16769731 PMC2806175

[ref106] Ledo A. Paul K. I. Burslem D. F. R. P. Ewel J. J. Barton C. Battaglia M.. (2018). Tree size and climatic water deficit control root to shoot ratio in individual trees globally. New Phytol. 217, 8–11. 10.1111/nph.14863, PMID: 29058312

[ref107] Li B. Li Y.-Y. Wu H.-M. Zhang F.-F. Li C.-J. Li X.-X.. (2016). Root exudates drive interspecific facilitation by enhancing nodulation and N(2) fixation. Proc. Natl. Acad. Sci. USA 113, 6496–6501. 10.1073/pnas.1523580113, PMID: 27217575 PMC4988560

[ref108] Li J. Zhu S. Song X. Shen Y. Chen H. Yu J.. (2006). A rice glutamate receptor-like gene is critical for the division and survival of individual cells in the root apical meristem. Plant Cell 18, 340–349. 10.1105/tpc.105.037713, PMID: 16377757 PMC1356543

[ref109] Liese R. Lübbe T. Albers N. W. Meier I. C. (2018). The mycorrhizal type governs root exudation and nitrogen uptake of temperate tree species. Tree Physiol. 38, 83–95. 10.1093/treephys/tpx131, PMID: 29126247

[ref110] Liu J. Samac D. A. Bucciarelli B. Allan D. L. Vance C. P. (2005). Signaling of phosphorus deficiency-induced gene expression in white lupin requires sugar and phloem transport. Plant J. 41, 257–268. 10.1111/j.1365-313X.2004.02289.x 15634202

[ref111] Liu J. Vance C. P. (2010). Crucial roles of sucrose and microRNA399 in systemic signaling of P deficiency: a tale of two team players? Plant Signal. Behav. 5, 1556–1560. 10.4161/psb.5.12.13293 21139425 PMC3115102

[ref112] López-Bucio J. Cruz-Ramırez A. Herrera-Estrella L. (2003). The role of nutrient availability in regulating root architecture. Curr. Opin. Plant Biol. 6, 280–287. 10.1016/S1369-5266(03)00035-9, PMID: 12753979

[ref113] Lucas García J. Barbas C. Probanza A. Barrientos M. Gutierrez Mañero F. (2001). Low molecular weight organic acids and fatty acids in root exudates of two Lupinus cultivars at flowering and fruiting stages. Phytochem. Anal. 12, 305–311. 11705257 10.1002/pca.596

[ref114] Lynch J. (1995). Root architecture and plant productivity. Plant Physiol. 109:7. 10.1104/pp.109.1.7, PMID: 12228579 PMC157559

[ref115] Lyu Y. Tang H. L. Li H. G. Zhang F. S. Rengel Z. Whalley W. R.. (2016). Major crop species show differential balance between root morphological and physiological responses to variable phosphorus supply. Front. Plant Sci. 7:1939. 10.3389/fpls.2016.01939, PMID: 28066491 PMC5174099

[ref116] Ma H.-K. Pineda A. van der Wurff A. W. G. Raaijmakers C. Bezemer T. M. (2017). Plant-soil feedback effects on growth, defense and susceptibility to a soil-borne disease in a cut flower crop: species and functional group effects. Front. Plant Sci. 8, 2127–2127. 10.3389/fpls.2017.02127 29312387 PMC5742127

[ref117] Ma J. F. Ryan P. R. Delhaize E. (2001). Aluminium tolerance in plants and the complexing role of organic acids. Trends Plant Sci. 6, 273–278. 10.1016/S1360-1385(01)01961-6, PMID: 11378470

[ref118] Mahmood T. Woitke M. Gimmler H. Kaiser W. M. (2002). Sugar exudation by roots of kallar grass [*Leptochloa fusca* (L.) Kunth] is strongly affected by the nitrogen source. Planta 214, 887–894. 10.1007/s00425-001-0697-x, PMID: 11941465

[ref119] Manck-Götzenberger J. Requena N. (2016). Arbuscular mycorrhiza symbiosis induces a major transcriptional reprogramming of the potato SWEET sugar transporter family. Front. Plant Sci. 7:487. 10.3389/fpls.2016.00487 27148312 PMC4830831

[ref120] Mariotte P. Mehrabi Z. Bezemer T. M. De Deyn G. B. Kulmatiski A. Drigo B. (2018). Plant-soil feedback: bridging natural and agricultural sciences. Trends Ecol. Evol. 33, 129–142. 10.1016/j.tree.2017.11.005 29241940

[ref121] Martin F. M. Uroz S. Barker D. G. (2017). Ancestral alliances: plant mutualistic symbioses with fungi and bacteria. Science 356:eaad4501. 10.1126/science.aad4501, PMID: 28546156

[ref122] McCormack M. L. Dickie I. A. Eissenstat D. M. Fahey T. J. Fernandez C. W. Guo D. (2015). Redefining fine roots improves understanding of below-ground contributions to terrestrial biosphere processes. New Phytol. 207, 505–518. 10.1111/nph.13363 25756288

[ref123] McCully M. E. Canny M. J. (1985). Localisation of translocated 14C in roots and root exudates of field-grown maize. Physiol. Plant. 65, 380–392. 10.1111/j.1399-3054.1985.tb08661.x

[ref124] Meharg A. Killham K. (1995). Loss of exudates from the roots of perennial ryegrass inoculated with a range of micro-organisms. Plant Soil 170, 345–349. 10.1007/BF00010488

[ref125] Meier I. C. Finzi A. C. Phillips R. P. (2017). Root exudates increase N availability by stimulating microbial turnover of fast-cycling N pools. Soil Biol. Biochem. 106, 119–128. 10.1016/j.soilbio.2016.12.004

[ref126] Meier I. C. Pritchard S. G. Brzostek E. R. McCormack M. L. Phillips R. P. (2015). The rhizosphere and hyphosphere differ in their impacts on carbon and nitrogen cycling in forests exposed to elevated CO_2_. New Phytol. 205, 1164–1174. 10.1111/nph.13122, PMID: 25348688

[ref127] Mencuccini M. Hölttä T. (2010). The significance of phloem transport for the speed with which canopy photosynthesis and belowground respiration are linked. New Phytol. 185, 189–203. 10.1111/j.1469-8137.2009.03050.x, PMID: 19825019

[ref128] Meyer S. De Angeli A. Fernie A. R. Martinoia E. (2010). Intra- and extra-cellular excretion of carboxylates. Trends Plant Sci. 15, 40–47. 10.1016/j.tplants.2009.10.002 19913451

[ref129] Moe L. A. (2013). Amino acids in the rhizosphere: from plants to microbes. Am. J. Bot. 100, 1692–1705. 10.3732/ajb.1300033, PMID: 23956051

[ref130] Mommer L. Kirkegaard J. van Ruijven J. (2016). Root-root interactions: towards a rhizosphere framework. Trends Plant Sci. 21, 209–217. 10.1016/j.tplants.2016.01.009, PMID: 26832947

[ref131] Mora-Macias J. Ojeda-Rivera J. O. Gutierrez-Alanis D. Yong-Villalobos L. Oropeza-Aburto A. Raya-Gonzalez J.. (2017). Malate-dependent Fe accumulation is a critical checkpoint in the root developmental response to low phosphate. Proc. Natl. Acad. Sci. USA 114, E3563–E3572. 10.1073/pnas.1701952114, PMID: 28400510 PMC5410833

[ref132] Mora-Macías J. Ojeda-Rivera J. O. Gutiérrez-Alanís D. Yong-Villalobos L. Oropeza-Aburto A. Raya-González J.. (2017). Malate-dependent Fe accumulation is a critical checkpoint in the root developmental response to low phosphate. Proc. Natl. Acad. Sci. 114, E3563–E3572. 10.1073/pnas.1701952114, PMID: 28400510 PMC5410833

[ref133] Münch E. (1930). Die stoffbewegungen in der Pflanze. Jena: Carl Fischer.

[ref134] Nacry P. Bouguyon E. Gojon A. (2013). Nitrogen acquisition by roots: physiological and developmental mechanisms ensuring plant adaptation to a fluctuating resource. Plant Soil 370, 1–29. 10.1007/s11104-013-1645-9

[ref135] Nadira U. A. Ahmed I. M. Wu F. Zhang G. (2016). The regulation of root growth in response to phosphorus deficiency mediated by phytohormones in a Tibetan wild barley accession. Acta Physiol. Plant. 38, 105. 10.1007/s11738-016-2124-8

[ref136] Naseer S. Lee Y. Lapierre C. Franke R. Nawrath C. Geldner N. (2012). Casparian strip diffusion barrier in Arabidopsis is made of a lignin polymer without suberin. Proc. Natl. Acad. Sci. 109, 10101–10106. 10.1073/pnas.1205726109, PMID: 22665765 PMC3382560

[ref137] Näsholm T. Kielland K. Ganeteg U. (2009). Uptake of organic nitrogen by plants. New Phytol. 182, 31–48. 10.1111/j.1469-8137.2008.02751.x, PMID: 19210725

[ref138] Nazoa P. Vidmar J. J. Tranbarger T. J. Mouline K. Damiani I. Tillard P.. (2003). Regulation of the nitrate transporter gene AtNRT2.1 in Arabidopsis thaliana: responses to nitrate, amino acids and developmental stage. Plant Mol. Biol. 52, 689–703. 10.1023/a:1024899808018, PMID: 12956537

[ref139] Nehls U. (2008). Mastering ectomycorrhizal symbiosis: the impact of carbohydrates. J. Exp. Bot. 59, 1097–1108. 10.1093/jxb/erm334, PMID: 18272925

[ref140] Ni J. Yu Z. Du G. Zhang Y. Taylor J. L. Shen C. (2016). Heterologous expression and functional analysis of rice glutamate receptor-like family indicates its role in glutamate triggered calcium flux in rice roots. Rice 9, 9. 10.1186/s12284-016-0081-x 26956369 PMC4783324

[ref141] Oburger E. Jones D. L. (2018). Sampling root exudates – mission impossible? Rhizosphere 6, 116–133. 10.1016/j.rhisph.2018.06.004

[ref142] Ogden M. Hoefgen R. Roessner U. Persson S. Khan G. (2018). Feeding the walls: how does nutrient availability regulate cell wall composition? Int. J. Mol. Sci. 19:2691. 10.3390/ijms19092691, PMID: 30201905 PMC6164997

[ref143] Ohkubo Y. Tanaka M. Tabata R. Ogawa-Ohnishi M. Matsubayashi Y. (2017). Shoot-to-root mobile polypeptides involved in systemic regulation of nitrogen acquisition. Nat. Plants. 3, 17029. 10.1038/nplants.2017.29, PMID: 28319056

[ref144] Okumoto S. Pilot G. (2011). Amino acid export in plants: a missing link in nitrogen cycling. Mol. Plant 4, 453–463. 10.1093/mp/ssr003, PMID: 21324969 PMC3143828

[ref145] Ortíz-Castro R. Contreras-Cornejo H. A. Macías-Rodríguez L. López-Bucio J. (2009). The role of microbial signals in plant growth and development. Plant Signal. Behav. 4, 701–712. 19820333 10.4161/psb.4.8.9047 PMC2801380

[ref146] Otani T. Ae N. Tanaka H. (1996). Phosphorus (P) uptake mechanisms of crops grown in soils with low P status. II. Significance of organic acids in root exudates of pigeonpea. Soil Sci. Plant Nutr. 42, 553–560. 10.1080/00380768.1996.10416324

[ref147] Padgett P. E. Leonard R. T. (1996). Free amino acid levels and the regulation of nitrate uptake in maize cell suspension cultures. J. Exp. Bot. 47, 871–883. 10.1093/jxb/47.7.871

[ref148] Pal’ove-Balang P. Mistrík I. (2002). Control of nitrate uptake by phloem-translocated glutamine in *Zea mays* L. seedlings. Plant Biol. 4, 440–445. 10.1055/s-2002-34123

[ref149] Parniske M. (2008). Arbuscular mycorrhiza: the mother of plant root endosymbioses. Nat. Rev. Microbiol. 6, 763. 10.1038/nrmicro1987, PMID: 18794914

[ref150] Paterson E. Sim A. Davidson J. Daniell T. J. (2016). Arbuscular mycorrhizal hyphae promote priming of native soil organic matter mineralisation. Plant Soil 408, 243–254. 10.1007/s11104-016-2928-8

[ref151] Paynel F. Murray P. J. Bernard Cliquet J. (2001). Root exudates: a pathway for short-term N transfer from clover and ryegrass. Plant Soil 229, 235–243. 10.1023/a:1004877214831

[ref152] Péret B. Clément M. Nussaume L. Desnos T. (2011). Root developmental adaptation to phosphate starvation: better safe than sorry. Trends Plant Sci. 16, 442–450. 10.1016/j.tplants.2011.05.006 21684794

[ref153] Phillips D. A. Fox T. C. King M. D. Bhuvaneswari T. V. Teuber L. R. (2004). Microbial products trigger amino acid exudation from plant roots. Plant Physiol. 136, 2887–2894. 10.1104/pp.104.044222, PMID: 15347793 PMC523350

[ref154] Pierik R. Mommer L. Voesenek L. A. C. J. (2013). Molecular mechanisms of plant competition: neighbour detection and response strategies. Funct. Ecol. 27, 841–853. 10.1111/1365-2435.12010

[ref155] Pii Y. Mimmo T. Tomasi N. Terzano R. Cesco S. Crecchio C. (2015a). Microbial interactions in the rhizosphere: beneficial influences of plant growth-promoting rhizobacteria on nutrient acquisition process. A review. Biol. Fertil. Soils 51, 403–415. 10.1007/s00374-015-0996-1

[ref156] Pii Y. Penn A. Terzano R. Crecchio C. Mimmo T. Cesco S. (2015b). Plant-microorganism-soil interactions influence the Fe availability in the rhizosphere of cucumber plants. Plant Physiol. Biochem. 87, 45–52. 10.1016/j.plaphy.2014.12.014 25544744

[ref157] Png G. K. Lambers H. Kardol P. Turner B. L. Wardle D. A. Laliberté E. (2019). Biotic and abiotic plant–soil feedback depends on nitrogen-acquisition strategy and shifts during long-term ecosystem development. Journal of Ecology 107, 142–153. 10.1111/1365-2745.13048

[ref158] Pratelli R. Voll L. M. Horst R. J. Frommer W. B. Pilot G. (2010). Stimulation of nonselective amino acid export by glutamine dumper proteins. Plant Physiol. 152, 762–773. 10.1104/pp.109.151746, PMID: 20018597 PMC2815850

[ref159] Prikryl Z. Vancura V. (1980). Root exudates of plants. 6. Wheat root exudation as dependent on growth concentration gradient of exudates and the presence of bacteria. Plant Soil 57, 69–83.

[ref160] Ramesh S. A. Tyerman S. D. Xu B. Bose J. Kaur S. Conn V.. (2015). GABA signalling modulates plant growth by directly regulating the activity of plant-specific anion transporters. Nat. Commun. 6:7879. 10.1038/ncomms8879, PMID: 26219411 PMC4532832

[ref161] Reid D. E. Ferguson B. J. Gresshoff P. M. (2011a). Inoculation- and nitrate-induced CLE peptides of soybean control NARK-dependent nodule formation. Mol. Plant-Microbe Interact. 24, 606–618. 10.1094/mpmi-09-10-0207 21198362

[ref162] Reid D. E. Ferguson B. J. Hayashi S. Lin Y. -H. Gresshoff P. M. (2011b). Molecular mechanisms controlling legume autoregulation of nodulation. Ann. Bot. 108, 789–795. 10.1093/aob/mcr205 21856632 PMC3177682

[ref163] Reinhart K. O. (2012). The organization of plant communities: negative plant-soil feedbacks and semiarid grasslands. Ecology 93, 2377–2385. 10.1890/12-0486.1, PMID: 23236909

[ref164] Rolland F. Baena-Gonzalez E. Sheen J. (2006). Sugar sensing and signaling in plants: conserved and novel mechanisms. Annu. Rev. Plant Biol. 57, 675–709. 10.1146/annurev.arplant.57.032905.105441 16669778

[ref165] Ross-Elliott T. J. Jensen K. H. Haaning K. S. Wager B. M. Knoblauch J. Howell A. H.. (2017). Phloem unloading in Arabidopsis roots is convective and regulated by the phloem-pole pericycle. elife 6:e24125. 10.7554/eLife.24125, PMID: 28230527 PMC5365319

[ref166] Rothstein D. E. (2009). Soil amino-acid availability across a temperate-forest fertility gradient. Biogeochemistry 92, 201–215. 10.1007/s10533-009-9284-1

[ref167] Rouached H. Arpat A. B. Poirier Y. (2010). Regulation of phosphate starvation responses in plants: signaling players and cross-talks. Mol. Plant 3, 288–299. 10.1093/mp/ssp120, PMID: 20142416

[ref168] Roy S. J. Gilliham M. Berger B. Essah P. A. Cheffings C. Miller A. J. (2008). Investigating glutamate receptor-like gene co-expression in *Arabidopsis thaliana*. Plant Cell Environ. 31, 861–871. 10.1111/j.1365-3040.2008.01801.x 18284583

[ref169] Rutschow H. L. Baskin T. I. Kramer E. M. (2011). Regulation of solute flux through plasmodesmata in the root meristem. Plant Physiol. 155, 1817–1826. 10.1104/pp.110.168187, PMID: 21325566 PMC3091107

[ref170] Ryan P. R. James R. A. Weligama C. Delhaize E. Rattey A. Lewis D. C. (2014). Can citrate efflux from roots improve phosphorus uptake by plants? Testing the hypothesis with near-isogenic lines of wheat. Physiol. Plant. 151, 230–242. 10.1111/ppl.12150 24433537

[ref171] Sasse J. Martinoia E. Northen T. (2018). Feed your friends: do plant exudates shape the root microbiome? Trends Plant Sci. 23, 25–41. 10.1016/j.tplants.2017.09.003 29050989

[ref172] Savage J. A. Clearwater M. J. Haines D. F. Klein T. Mencuccini M. Sevanto S. (2016). Allocation, stress tolerance and carbon transport in plants: how does phloem physiology affect plant ecology? Plant Cell Environ. 39, 709–725. 10.1111/pce.12602 26147312

[ref173] Schimel J. P. Bennett J. (2004). Nitrogen mineralization: challenges of a changing paradigm. Ecology 85, 591–602. 10.1890/03-8002

[ref174] Schmidt H. Nunan N. Höck A. Eickhorst T. Kaiser C. Woebken D. (2018). Recognizing patterns: spatial analysis of observed microbial colonization on root surfaces. Front. Environ. Sci. 6:61. 10.3389/fenvs.2018.00061

[ref175] Scott-Denton L. E. Rosenstiel T. N. Monson R. K. (2006). Differential controls by climate and substrate over the heterotrophic and rhizospheric components of soil respiration. Glob. Chang. Biol. 12, 205–216. 10.1111/j.1365-2486.2005.01064.x

[ref176] Semchenko M. Hutchings M. J. John E. A. (2007). Challenging the tragedy of the commons in root competition: confounding effects of neighbour presence and substrate volume. J. Ecol. 95, 252–260. 10.1111/j.1365-2745.2007.01210.x

[ref177] Shane M. W. Lambers H. (2005). Cluster roots: a curiosity in context. Plant Soil 274, 101–125. 10.1007/s11104-004-2725-7

[ref178] Shi S. Richardson A. E. O'Callaghan M. DeAngelis K. M. Jones E. E. Stewart A.. (2011). Effects of selected root exudate components on soil bacterial communities. FEMS Microbiol. Ecol. 77, 600–610. 10.1111/j.1574-6941.2011.01150.x, PMID: 21658090

[ref179] Shishkova S. Rost T. L. Dubrovsky J. G. (2008). Determinate root growth and meristem maintenance in angiosperms. Ann. Bot. 101, 319–340. 10.1093/aob/mcm251, PMID: 17954472 PMC2701811

[ref180] Smith S. E. Read D. J. (2008). Mycorrhizal symbiosis. Amsterdam: Academic Press.

[ref181] Smith S. E. Smith F. A. (2011). Roles of arbuscular mycorrhizas in plant nutrition and growth: new paradigms from cellular to ecosystem scales. Annu. Rev. Plant Biol. 62, 227–250. 10.1146/annurev-arplant-042110-103846 21391813

[ref182] Somssich M. Khan G. A. Persson S. (2016). Cell wall heterogeneity in root development of arabidopsis. Front. Plant Sci. 7:1242. 10.3389/fpls.2016.01242 27582757 PMC4987334

[ref183] Steinauer K. Chatzinotas A. Eisenhauer N. (2016). Root exudate cocktails: the link between plant diversity and soil microorganisms? Ecol. Evol. 6, 7387–7396. 10.1002/ece3.2454, PMID: 28725406 PMC5513276

[ref184] Ström L. Owen A. G. Godbold D. L. Jones D. L. (2002). Organic acid mediated P mobilization in the rhizosphere and uptake by maize roots. Soil Biol. Biochem. 34, 703–710. 10.1016/S0038-0717(01)00235-8

[ref185] Sun C.-H. Yu J.-Q. Hu D. -G. (2017). Nitrate: a crucial signal during lateral roots development. Front. Plant Sci. 8, 485. 10.3389/fpls.2017.00485, PMID: 28421105 PMC5379155

[ref186] Svistoonoff S. Creff A. Reymond M. Sigoillot-Claude C. Ricaud L. Blanchet A. (2007). Root tip contact with low-phosphate media reprograms plant root architecture. Nat. Genet. 39, 792–796. http://www.nature.com/ng/journal/v39/n6/suppinfo/ng2041_S1.html 17496893 10.1038/ng2041

[ref187] Tapken D. Anschutz U. Liu L. H. Huelsken T. Seebohm G. Becker D. (2013). A plant homolog of animal glutamate receptors is an ion channel gated by multiple hydrophobic amino acids. Sci. Signal. 6:279, ra47. 10.1126/scisignal.2003762 23757024

[ref188] Teardo E. Formentin E. Segalla A. Giacometti G. M. Marin O. Zanetti M.. (2011). Dual localization of plant glutamate receptor AtGLR3.4 to plastids and plasmamembrane. Biochim. Biophys. Acta 1807 359–367. 10.1016/j.bbabio.2010.11.008, PMID: 21110940

[ref189] Tegeder M. Masclaux-Daubresse C. (2018). Source and sink mechanisms of nitrogen transport and use. New Phytol. 217, 35–53. 10.1111/nph.14876, PMID: 29120059

[ref190] Teste F. P. Kardol P. Turner B. L. Wardle D. A. Zemunik G. Renton M.. (2017). Plant-soil feedback and the maintenance of diversity in Mediterranean-climate shrublands. Science 355, 173–176. 10.1126/science.aai8291, PMID: 28082588

[ref191] Thirkell T. J. Charters M. D. Elliott A. J. Sait S. M. Field K. J. (2017). Are mycorrhizal fungi our sustainable saviours? Considerations for achieving food security. J. Ecol. 105, 921–929. 10.1111/1365-2745.12788

[ref192] Ticconi C. A. Abel S. (2004). Short on phosphate: plant surveillance and countermeasures. Trends Plant Sci. 9, 548–555. 10.1016/j.tplants.2004.09.003 15501180

[ref193] Tillard P. Passama L. Gojon A. (1998). Are phloem amino acids involved in the shoot to root control of NO^−3^ uptake in *Ricinus communis* plants? J. Exp. Bot. 49, 1371–1379. 10.1093/jxb/49.325.1371

[ref194] Toljander J. F. Lindahl B. D. Paul L. R. Elfstrand M. Finlay R. D. (2007). Influence of arbuscular mycorrhizal mycelial exudates on soil bacterial growth and community structure. FEMS Microbiol. Ecol. 61, 295–304. 10.1111/j.1574-6941.2007.00337.x, PMID: 17535297

[ref195] Tsikou D. Yan Z. Holt D. B. Abel N. B. Reid D. E. Madsen L. H.. (2018). Systemic control of legume susceptibility to rhizobial infection by a mobile microRNA. Science 362, 233–236. 10.1126/science.aat6907, PMID: 30166437

[ref196] Valentinuzzi F. Cesco S. Tomasi N. Mimmo T. (2015). Influence of different trap solutions on the determination of root exudates in *Lupinus albus* L. Biol. Fertil. Soils 51, 757–765. 10.1007/s00374-015-1015-2

[ref197] Van Der Heijden M. G. Martin F. M. Selosse M. A. Sanders I. R. (2015). Mycorrhizal ecology and evolution: the past, the present, and the future. New Phytol. 205, 1406–1423. 10.1111/nph.13288, PMID: 25639293

[ref198] Van Nuland M. E. Bailey J. K. Schweitzer J. A. (2017). Divergent plant-soil feedbacks could alter future elevation ranges and ecosystem dynamics. Nat. Ecol. Evol. 1, 0150. 10.1038/s41559-017-0150, PMID: 28812635

[ref199] Verbon E. H. Liberman L. M. (2016). Beneficial microbes affect endogenous mechanisms controlling root development. Trends Plant Sci. 21, 218–229. 10.1016/j.tplants.2016.01.013 26875056 PMC4772406

[ref200] Vidal A. Hirte J. Bender S. F. Mayer J. Gattinger A. Höschen C. (2018). Linking 3D soil structure and plant-microbe-soil carbon transfer in the rhizosphere. Front. Environ. Sci. 6:9. 10.3389/fenvs.2018.00009

[ref201] Vincill E. D. Bieck A. M. Spalding E. P. (2012). Ca^2+^ conduction by an amino acid-gated ion channel related to glutamate receptors. Plant Physiol. 159, 40–46. 10.1104/pp.112.197509, PMID: 22447719 PMC3375973

[ref202] Vincill E. D. Clarin A. E. Molenda J. N. Spalding E. P. (2013). Interacting glutamate receptor-like proteins in phloem regulate lateral root initiation in Arabidopsis. Plant Cell 25, 1304–1313. 10.1105/tpc.113.110668, PMID: 23590882 PMC3663269

[ref203] Vranova V. Rejsek K. Skene K. R. Janous D. Formanek P. (2013). Methods of collection of plant root exudates in relation to plant metabolism and purpose: a review. J. Plant Nutr. Soil Sci. 176, 175–199. 10.1002/jpln.201000360

[ref204] Walch-Liu P. Liu L.-H. Remans T. Tester M. Forde B. G. (2006a). Evidence that l-glutamate can act as an exogenous signal to modulate root growth and branching in *Arabidopsis thaliana*. Plant Cell Physiol. 47, 1045–1057. 10.1093/pcp/pcj075 16816406

[ref205] Walch-Liu P. I. A. Ivanov I. I. Filleur S. Gan Y. Remans T. Forde B. G. (2006b). Nitrogen regulation of root branching. Ann. Bot. 97, 875–881. 10.1093/aob/mcj601 16339770 PMC2803407

[ref206] Warren C. R. (2015). Wheat roots efflux a diverse array of organic N compounds and are highly proficient at their recapture. Plant Soil 397, 147–162. 10.1007/s11104-015-2612-4

[ref207] Warren C. R. (2016). Simultaneous efflux and uptake of metabolites by roots of wheat. Plant Soil 406, 359–374. 10.1007/s11104-016-2892-3

[ref208] Watt M. Evans J. R. (1999). Linking development and determinacy with organic acid efflux from proteoid roots of white lupin grown with low phosphorus and ambient or elevated atmospheric CO_2_ concentration. Plant Physiol. 120, 705–716. 10.1104/pp.120.3.705, PMID: 10398705 PMC59308

[ref209] Watt M. Hugenholtz P. White R. Vinall K. (2006). Numbers and locations of native bacteria on field-grown wheat roots quantified by fluorescence in situ hybridization (FISH). Environ. Microbiol. 8, 871–884. 10.1111/j.1462-2920.2005.00973.x, PMID: 16623744

[ref210] Williams L. E. Lemoine R. Sauer N. (2000). Sugar transporters in higher plants – a diversity of roles and complex regulation. Trends Plant Sci. 5, 283–290. 10.1016/S1360-1385(00)01681-2 10871900

[ref211] Wubs E. R. J. Bezemer T. M. (2018). Plant community evenness responds to spatial plant-soil feedback heterogeneity primarily through the diversity of soil conditioning. Funct. Ecol. 32, 509–521. 10.1111/1365-2435.13017

[ref212] Yang H. Bogner M. Stierhof Y. -D. Ludewig U. (2010). H^+^-independent glutamine transport in plant root tips. PLoS One 5:e8917. 10.1371/journal.pone.0008917, PMID: 20111724 PMC2811748

[ref213] Yang N. J. Hinner M. J. (2015). Getting across the cell membrane: an overview for small molecules, peptides, and proteins. Methods Mol. Biol. 1266, 29–53. 10.1007/978-1-4939-2272-7_3 25560066 PMC4891184

[ref214] Yin H. Li Y. Xiao J. Xu Z. Cheng X. Liu Q. (2013). Enhanced root exudation stimulates soil nitrogen transformations in a subalpine coniferous forest under experimental warming. Glob. Chang. Biol. 19, 2158–2167. 10.1111/gcb.12161, PMID: 23504744

[ref215] Zhang L. Feng G. Declerck S. (2018). Signal beyond nutrient, fructose, exuded by an arbuscular mycorrhizal fungus triggers phytate mineralization by a phosphate solubilizing bacterium. ISME J. 12, 2339–2351. 10.1038/s41396-018-0171-4, PMID: 29899507 PMC6155042

[ref216] Zhu S. Vivanco J. M. Manter D. K. (2016). Nitrogen fertilizer rate affects root exudation, the rhizosphere microbiome and nitrogen-use-efficiency of maize. Appl. Soil Ecol. 107(Suppl. C), 324–333. 10.1016/j.apsoil.2016.07.009

[ref217] Zimmermann S. Ehrhardt T. Plesch G. Müller-Röber B. (1999). Ion channels in plant signaling. Cell. Mol. Life Sci. 55, 183–203. 10.1007/s000180050284, PMID: 24481913 PMC11146866

