# Corrigendum: Root Exudation of Primary Metabolites: Mechanisms and Their Roles in Plant Responses to Environmental Stimuli

**DOI:** 10.3389/fpls.2019.00420

**Published:** 2019-04-09

**Authors:** Alberto Canarini, Christina Kaiser, Andrew Merchant, Andreas Richter, Wolfgang Wanek

**Affiliations:** ^1^Terrestrial Ecosystem Research, Department of Microbiology and Ecosystem Science, Research Network ‘Chemistry Meets Microbiology’, University of Vienna, Vienna, Austria; ^2^Faculty of Science, Sydney Institute of Agriculture, The University of Sydney, Sydney, NSW, Australia

**Keywords:** root exudates, soil micro-organisms, root architecture, nutrient sensing, priming effect, mycorrhiza

In the original article, there was a mistake in Figure 1 as published. The group of cells highlighted in red (in the upper part of the figure) are named “meristem,” while it should more specifically refer to the “quiescent center.” The corrected Figure 1 appears below.

**Figure 1:**
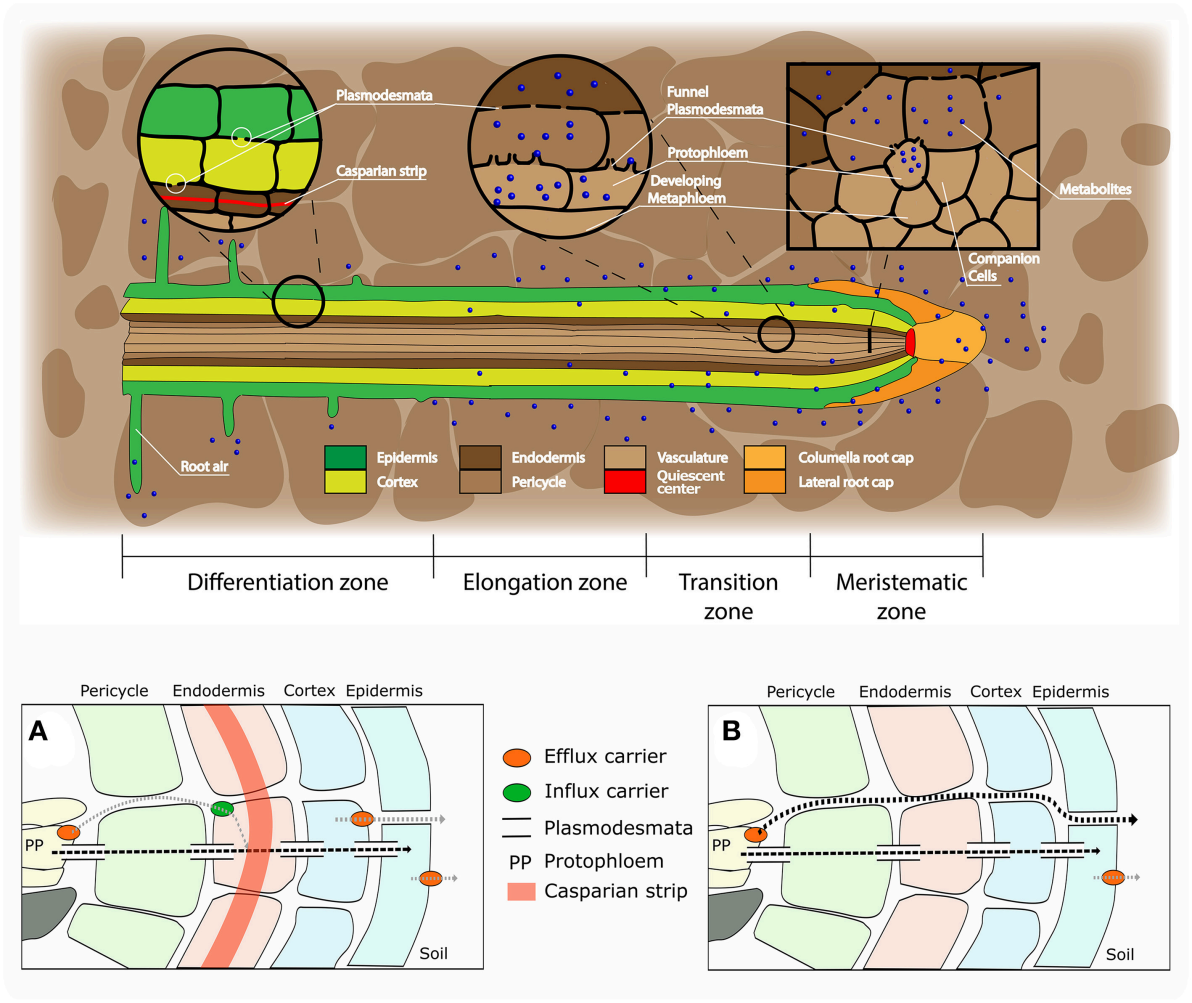
Root structure and areas of root exudation. The upper figure represents the longitudinal section of a root. Tissues are indicated in different colors for the different zones of the root (listed at the bottom). The two circles focus on two distinct zones, a differentiated vs. an undifferentiated area, to show the presence of a Casparian strip and low abundance of plasmodesmata in the differentiated area (left circle), and the presence of funnel plasmodesmata in the undifferentiated area (right circle). The square represents a cross section close to the meristematic area where root exudation is the highest. The lower figures represent a schematic representation of solute movement sites from phloem unloading to the soil environment, either in the differentiation zone **(A)** or in the undifferentiated root tip **(B)**. **(A)** Solutes move both through the symplastic and apoplastic pathways, but then they are re-uptaken into the cytoplasm as the Casparian strip limits the apoplastic pathway. Only the cortex and epidermis are responsible for the flux of metabolites into the apoplast and consecutively into the soil (root exudation). Cortex and epidermis represents the major control point for root exudation. **(B)** At the phloem unloading site, both symplastic and apoplastic pathways are used. Because of the lack of a Casparian strip solutes can move out of the root (root exudation) through both the apoplastic and the symplastic pathway.

The authors apologize for this error and state that this does not change the scientific conclusions of the article in any way. The original article has been updated.

